# The distinct effects of *P18* overexpression on different stages of hematopoiesis involve TGF-β and NF-κB signaling

**DOI:** 10.1038/s41598-021-03263-2

**Published:** 2021-12-14

**Authors:** Danying Yi, Lijiao Zhu, Yuanling Liu, Jiahui Zeng, Jing Chang, Wencui Sun, Jiawen Teng, Yonggang Zhang, Yong Dong, Xu Pan, Yijin Chen, Ya Zhou, Mowen Lai, Qiongxiu Zhou, Jiaxin Liu, Bo Chen, Feng Ma

**Affiliations:** 1 Center for Stem Cell Research and Application, Institute of Blood Transfusion, Chinese Academy of Medical Sciences & Peking Union Medical College (CAMS & PUMC), Chengdu, 610052 China; 2 State Key Laboratory of Experimental Hematology, CAMS & PUMC, Tianjin, 300020 China

**Keywords:** Gene regulation, Stem cells, Haematopoietic stem cells, Stem-cell differentiation

## Abstract

Deficiency of *P18* can significantly improve the self-renewal potential of hematopoietic stem cells (HSC) and the success of long-term engraftment. However, the effects of *P18* overexpression, which is involved in the inhibitory effects of *RUNX1b* at the early stage of hematopoiesis, have not been examined in detail. In this study, we established inducible *P18*/hESC lines and monitored the effects of *P18* overexpression on hematopoietic differentiation. Induction of *P18* from day 0 (D0) dramatically decreased production of CD34^high^CD43− cells and derivative populations, but not that of CD34^low^CD43− cells, changed the cell cycle status and apoptosis of KDR+ cells and downregulated the key hematopoietic genes at D4, which might cause the severe blockage of hematopoietic differentiation at the early stage. By contrast, induction of *P18* from D10 dramatically increased production of classic hematopoietic populations and changed the cell cycle status and apoptosis of CD45+ cells at D14. These effects can be counteracted by inhibition of TGF-β or NF-κB signaling respectively. This is the first evidence that *P18* promotes hematopoiesis, a rare property among cyclin-dependent kinase inhibitors (CKIs).

## Introduction

Hematopoiesis can be divided into primitive and definitive hematopoiesis, which originate from the yolk sac (YS) and the aorta/gonad/mesonephros (AGM) region, respectively^1–4^. Cell differentiation, including development of the hematopoietic lineage, is closely related to the cell cycle and is regulated by multiple cell cycle factors^5,6^. Among those factors, cyclin-dependent kinase inhibitors (CKIs) can manipulate the cell cycle and induce cell cycle arrest^7^. CKIs can be divided into two families: the Cip/Kip family, including p21Cip1/Waf1/Sdi1 (*P21*), p27Kip1 (*P27*), and p57Kip2 (*P57*), and the INK4 family, including p16INK4a (*P16*), p15INK4b (*P15*), p18INK4c (*P18*), and p19INK4d.2 (*P19*). *P15*, *P18*, *P19*, and *P27* are expressed in all cell lines^8^. Members of the INK4 family play important roles in hematopoietic differentiation, which is downstream of the TGF-β/SMAD signaling pathway and is also controlled by *RUNX1*, especially in adulthood^9–11^.

Among the members of the INK4 family, p18INK4c (cyclin-dependent kinase inhibitor 2C, *CDKN2C*) is focally expressed during embryonic development^12,13^ and has important functions during hematopoiesis^14,15^. By interacting with the Cyclin D–CDK4/CDK6 complex, p18INK4c blocks the activation of the CDK kinases, thus playing a key role in controlling the G1 phase of the cell cycle. This interaction is associated with G1 arrest^16^.

Deletion or inhibition of the *P18* gene can boost the self-renewal potential of hematopoietic stem cells and strikingly improve long-term engraftment by increasing self-renewal and differentiation of primitive hematopoietic cells in murine transplant models^16–18^. In comparison with the wild-type HSCs of unmanipulated young mice, *P18*-deficient HSCs can maintain their competitiveness and retain the potential for multi-lineage differentiation after multiple rounds of continuous bone marrow transplantation for as long as 3 years^16^. In addition, deletion of *P18* significantly slows hematopoietic exhaustion caused by deletion of *P21*^19^. Relative to other CKIs, such as *P21, P27*, and *P16*, deletion of *P18* has a stronger pro-hematopoietic effect, increasing the self-renewal time of hematopoietic stem cells and obtaining the advantages of transplantation^20,21^. Proliferation of hematopoietic progenitor cells (HPCs) is reduced in *P18*−/− mouse bone marrow, indicating that deletion of *P18* has a positive effect on HPCs in vivo^22^. To date, however, the effects of *P18* overexpression on hematopoiesis have not been systematically explored.

In previous research, we found that overexpression of *RUNX1b* during early hematopoiesis prevents the generation of CD34+ cells^1^. Subsequent studies revealed that *RUNX1b* overexpression also changes the status of the cell cycle and increases the expression of some cell cycle regulators, including *P18* (unpublished data). Hence, we sought to elucidate the function of *P18* on different stages of hematopoiesis using a mature inducible expression system based on *piggy*Bac transposon and the AGM-S3 co-culture system that we established previously^1,23,24^. Our findings provide the first evidence that *P18* overexpression can promote hematopoiesis, providing insight into the molecular mechanisms underlying CKI activity during hematopoietic differentiation.

## Material and methods

### Co-culture of hESCs with AGM-S3 cells

This study was approved by the institutional ethics committee of Institute of Blood Transfusion, Chinese Academy of Medical Sciences and Peking Union Medical College (CAMS & PUMC). AGM-S3 cells (provided by Prof. Tatsutoshi Nakahata) were plated in 12-well plates at 1 × 10^5^ cells per well and cultured in an incubator containing 5% CO_2_ at 37 °C. After the cells had grown to 80–100% confluence, they were irradiated with 13 Gy of X-rays. Undifferentiated hESCs (provided by Prof. Tao Cheng) were cut into small squares containing 0.5–1 × 10^3^ cells each by 200 μl tips, which were inoculated into 12-well plates (25 pieces per well). hPSC maintenance medium (Dulbecco’s modified Eagle’s medium (DMEM) with high glucose, F-12 nutrient mixture, 20% knockout serum replacement (KSR; Gibco), 1% L-glutamine, 1% non-essential amino acid solution (NEAA; Gibco), and 5 ng/ml basic FGF(b-FGF; Wako)) was used for the co-culture system for the first 3 days, and then replaced with hematopoiesis-inducing medium (Iscove’s modified Dulbecco’s medium (IMDM) containing 10% fetal bovine serum (FBS; Hyclone), 1% NEAA (Gibco), 60 ng/ml ascorbic acid (Sigma), and 20 ng/ml vascular endothelial growth factor (VEGF; Wako)); this day was defined as D0. The co-cultures were grown for up to 14 days with 5% CO_2_ at 37 °C, with a medium replacement once per day. The detailed procedure was defined previously^1,23,24^.

### Confirming the inhibitory effects of P18 overexpression and the antagonistic effects of inhibition of TGF-β signaling at the early stage of hematopoiesis

The establishment of *P18* (*CDKN2C*) inducible hESC lines (referred as *P18*/hESCs) was described in “Supplemental materials and methods”. D0-induced *P18*/hESCs co-cultured with AGM-S3 cells were treated without induction or with DOX from D0, D2, D4, D6, D8, D10, D12, or with DOX and 0.33 μM RepSox (Selleck Inc, dissolved in DMSO) from D0, as previously described^1^. An equal volume of DMSO was added to control samples. Treated co-cultures were subjected to cell-cycle analysis at D4 and flow cytometry using 7-AAD and anti-CD34/CD43 antibodies (D8) or 7-AAD and anti-CD34/CD43/CD45, CD71/GPA, or CD34/CD43/GPA/CD41a antibodies (D14). Untreated co-cultures were used as negative controls. The detail information of flow cytometry was described in “Supplemental materials and methods”.

### Hematopoietic colony-forming assays

D14 *P18*/hESC co-cultured cells induced with DOX from D0, D6, or D10, or not induced, were dissociated into single cells with 0.25% trypsin–EDTA solution, centrifuged at 400*g* for 5 min, and resuspended in 400 μl of IMDM medium. After cell counting. 5 × 10^4^ cells in suspension were mixed well on methylcellulose (H4320, STEM CELL) containing 1% Antibiotic–Antimycotic (Gibco) and cytokines, as previously described^23–25^, and then 1.1 ml of the mixture was divided into each 35-mm Petri dishes at a final concentration of 5 × 10^4^ cells per dish. The cells were incubated for 12–14 days at 37 °C in a 5% CO_2_/95% humidity incubator. Colony forming unit–erythrocyte (CFU-E) was determined after 7 days, and other types of colonies were counted after 12–14 days.

### Further hematopoietic culture analysis

Non-induced or induced *P18*/hESC co-cultures at D2 or D6 were dissociated by treatment with 0.05% trypsin EDTA solution and stained with 7-AAD and anti-KDR antibodies (D2) or anti-CD34/CD43 (D6). KDR+ cells at D2 or CD34^high^CD43- and CD34^low^CD43- cells at D6 were sorted using a BD FACSJazz Cell Sorter, and their purity was confirmed by flow cytometry analysis. 1 × 10^4^ sorted KDR+ cells were re-plated on irradiated AGM-S3 cells in each well of a 24-well plate, and 1 × 10^4^ sorted CD34^high^CD43− and CD34^low^CD43− cells were re-plated in FLHD (full-lineage hematopoietic differentiation, IMDM containing 10% FBS, 100 ng/ml SCF, 100 ng/ml IL-6, 10 ng/ml IL-3, 10 ng/ml FL, 10 ng/ml TPO, and 4 IU/ml EPO) medium in each well of a 48-well plate, further cultured with or without DOX induction for 11 or 14 days respectively. Half of the media was replaced every day, and flow cytometry analysis was performed. The detail information of flow cytometry and sorting was described in “Supplemental materials and methods”.

### Confirming the activation of NF-κB signaling and the antagonistic effects of NF-κB signaling inhibition on P18 overexpression at the late stage of hematopoiesis

D10-induced *P18*/hESCs co-cultured with AGM-S3 cells were detected by qRT-PCR at D14 using primers for *NFKB1* and *NFKB2*. QNZ, an inhibitor of the NF-κB signaling pathway (Selleck Inc) was dissolved in DMSO; siRNAs against *NFKB1* (Sangon Biotech Inc, Shanghai, China) were described previously^23,24^. D10-induced *P18*/hESC co-cultures were treated from D10 with 20 nM QNZ (or an equal volume of DMSO) or 20 nM siRNA against *NFKB1* (siRNA NFKB-1 vs NFKB-3 = 1:1 mixture or an equal concentration of control siRNA), and changed fresh media every day. At D14, the cells were evaluated by qRT-PCR, flow cytometry, and cell cycle analysis; untreated co-cultures were used as negative controls. qPCR primer pairs are listed in Table S1. siRNA sequences are listed in Table S3. The detail information of cell cycle analysis and flow cytometry was described in “Supplemental materials and methods”.

### Statistical analysis

All experimental data are described as means ± SD, and statistical significance was evaluated using Student’s t-test. p < 0.05 was considered statistically significant. FlowJo 10 (https://www.flowjo.com/solutions/flowjo/downloads/) and GraphPad Prism5 (https://www.graphpad.com/scientific-software/prism/) were used for data analysis.

### Method statement

All methods were carried out in accordance with relevant guidelines and regulations.

## Results

### Overexpression of RUNX1b upregulates P18 in co-culture on D4

**Figure 1:**
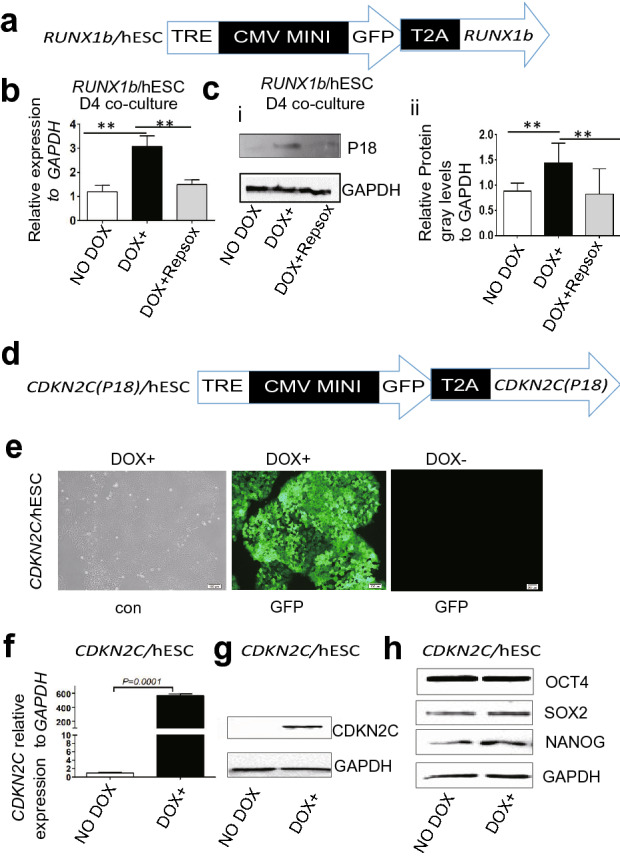
Involvement of *P18* is involved in inhibitory effects on the mesoderm–hemogenesis transition by *RUNX1b*, and establishment of inducible *P18* transgenic hESC lines. D0-induced *RUNX1b*/hESCs (**a**) were co-cultured with AGM-S3 cells at D4. (**b**) qRT-PCR analysis and (**c**) Western blotting (WB) analysis revealed that when DOX was added from D0, *P18* was upregulated at the mRNA and protein levels at D4, and that these effects can be counteracted by addition of 0.33 μM RepSox from D0. (**d**) Schematic diagram of the *piggy*Bac vector used to induce *P18* overexpression. TRE, tet-on regulation element; CMV Mini, minimum promoter of cytomegalovirus; T2A, *Thosea asigna* virus 2A peptide. (**e**) After *P18*/hESCs was treated with DOX for 48 h, co-expression of GFP was observed by fluorescence microscopy. (**f**) qRT-PCR analysis and (**g**) WB analysis confirmed that inducible expression of *P18* was highly stringent and effective at the mRNA and protein levels. GAPDH was used as an internal control. (**h**) WB analysis with anti-SOX2, -OCT4, and -NANOG antibodies confirmed the normal pluripotency of *P18*/hESCs. The loading control was GAPDH. All results are expressed as means ± SD of three repeated experiments, and p < 0.05 was considered significant (*p < 0.05, **p < 0.01, ***p < 0.001, ****p < 0.0001).

In co-cultures of *RUNX1b*/hESCs (Fig. 1a) with AGM-S3 cells, overexpression of *RUNX1b* at the early stage can block the mesoderm–hemogenesis transition, and treatment with 0.33 µM RepSox partially alleviates this blockage^1^. qRT-PCR (Fig. 1b) and western blotting (Fig. 1c) revealed that when *RUNX1b*/hESCs were induced at day 0 (D0), expression of *P18* was upregulated at D4 (see Experimental Procedures for the definition of D0). All of these effects can be counteracted by addition of 0.33 µM RepSox from D0 (Fig. 1b,c). Together, these results indicated that *P18* might be relevant to the inhibitory effects of *RUNX1b* on hematopoiesis.

### P18/hESCs exhibit inducible P18 overexpression and normal pluripotency

After *P18*/hESCs were induced with DOX for 48 h, fluorescence imaging, qRT-PCR, and western blotting confirmed that *P18* overexpression had been successfully achieved and stringently controlled (Fig. 1d–g). Western blotting revealed that OCT4, SOX2, and NANOG were normally expressed in *P18*/hESCs with or without DOX treatment, confirming that these cells had retained their normal pluripotency (Fig. 1h).

### CD34+CD43− can be obviously divided into CD34^low^CD43− and CD34^high^CD43− subpopulations with different traits during hematopoietic differentiation

**Figure 2:**
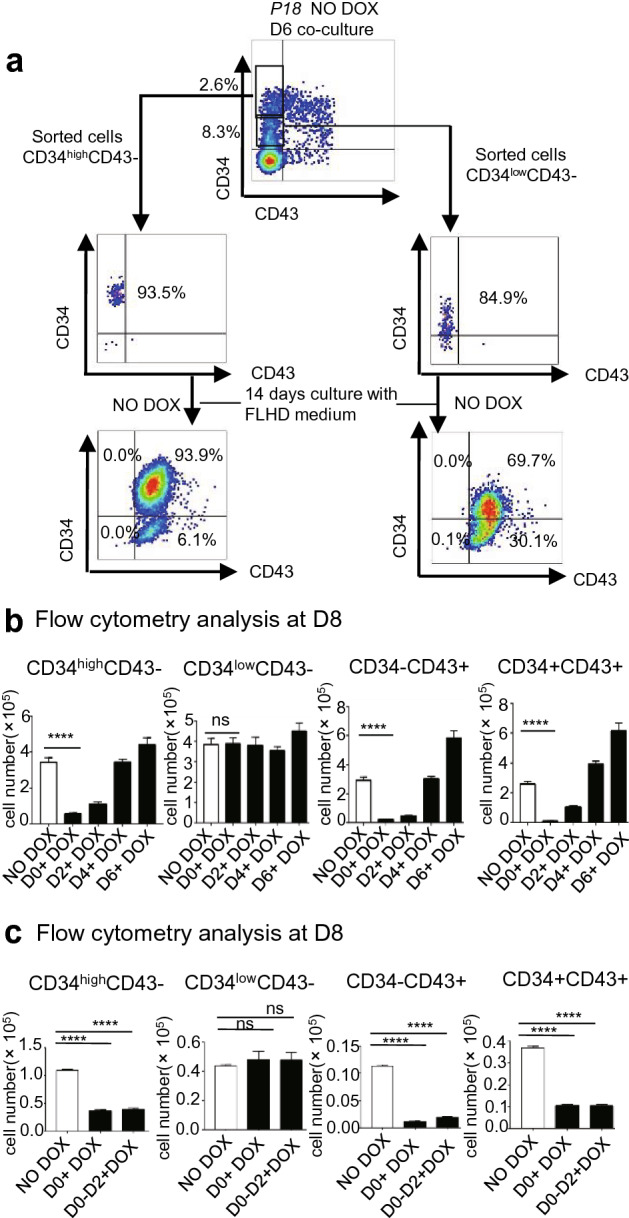
Overexpression of *P18* from D0 inhibits hematopoiesis in co-culture with AGM-S3 cells. (**a**) *P18*/hESCs were cultured with AGM-S3 cells, 1 × 10^4^ CD34^High^CD43− and CD34^Low^CD43− cells were sorted from non-induced co-cultures at D6, and then re–re-plated in FLHD medium in each well of a 48-well plate without DOX induction for 14 days. (**b**) *P18*/hESCs were treated with or without DOX from D0, D2, D4, or D6, and then subjected to flow cytometry at D8 using 7-AAD and anti-CD34/CD43 antibodies. The GFP+ fractions of non-induced co-cultures and of co-cultures treated with DOX were compared. Overexpression of *P18* at the early stage, especially from D0, led to significantly reduced production of CD34^high^CD43−, CD34−CD43+, and CD34+CD43+ cells at D8. (**c**) When *P18* was induced from D0 to D8 or only from D0 to D2, production of the CD34^high^CD43−, CD34−CD43+, and CD34+CD43+ populations were also reduced to a similar degree at D8. All results are expressed as means ± SD of the three repeated experiments, and p < 0.05 was considered significant (*p < 0.05, **p < 0.01, ***p < 0.001, ****p < 0.0001).

We found that CD34+CD43− cells can be clearly divided into two subpopulations: CD34^low^CD43− and CD34^high^CD43− cells. To investigate the difference in hematopoietic differentiation between them, these two subpopulations were sorted at D6, and subsequently resuspended in FLHD (full-lineage hematopoietic differentiation) medium without DOX induction (Fig. 2a). The results of flow cytometry analysis showed that compared with CD34^low^CD43-, in FLHD medium CD34^high^CD43- subpopulations can produce more CD34 + CD43 + cells, which expression level of CD34 protein was much higher.

### Overexpression of P18 at the early stage blocks hematopoietic differentiation and the transition from CD34^low^CD43− cells to CD34^high^CD43− cells

*P18*/hESCs treated with DOX starting from D0, D2, D4, or D6, or without DOX (as a negative control) were subjected to flow cytometry analysis at D8 to compare non-induced co-cultures and the GFP+ fractions of co-cultures treated with DOX. Overexpression of *P18* at the early stage, especially from D0, significantly decreased production of CD34^high^CD43−, CD34−CD43+, and CD34+CD43+ populations at D8; these effects gradually weakened when *P18* overexpression was initiated later, disappearing after D6 (Fig. 2b, Fig. S1a). When *P18* was induced from D0 to D8 or only from D0 to D2, production of these populations was reduced to similar degrees at D8 (Fig. 2c, Fig. S1b). By contrast, the CD34^low^CD43− population was not significantly affected. These results indicated that overexpression of *P18* at the early stage severely blocks development of CD34^high^CD43− and its derived populations, except for the CD34^low^CD43− population.

**Figure 3:**
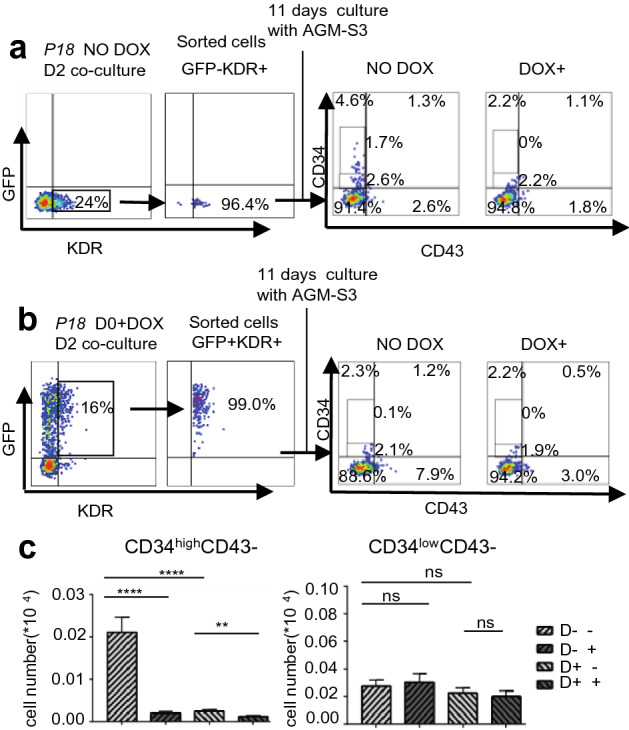
Overexpression of *P18* at the early stage blocks formation of CD34^high^CD43− cells but not CD34^low^CD43− cells. KDR+ cells were sorted from non-induced (**a**) or induced (**b**) *P18*/hESCs co-cultured with AGM-S3 at D2. About 1 × 10^4^ sorted cells were re-plated on irradiated AGM-S3 (24 wells) treated with or without DOX, and FACS analysis with 7-AAD and anti-CD34/CD43 antibody was performed after 11 days. (**c**) Production of CD34^high^CD43− cells was dramatically decreased by overexpression of *P18* from D0 to D2, whereas production of CD34^low^CD43− cells was not severely affected. All results were expressed as means ± SD of three repeated experiments, and p < 0.05 was considered significant (*p < 0.05, **p < 0.01, ***p < 0.001, ****p < 0.0001).

To investigate the inhibitory effects of *P18* overexpression on generation of CD34+ cells, we sorted KDR+ cells at D2 from non-induced or induced *P18*/hESCs co-cultured with AGM-S3, and subsequently cultured them on irradiated AGM-S3 cells with or without DOX (Fig. 3). Flow cytometry revealed that production of CD34^high^CD43− cells was significantly decreased by overexpression of *P18* from D0 to D2, whereas production of CD34^low^CD43− cells was not significantly affected.

### Inhibition of TGF-β signaling counteracts the inhibitory effects of P18 overexpression at the early stage and alters cell-cycle status and apoptosis

**Figure 4:**
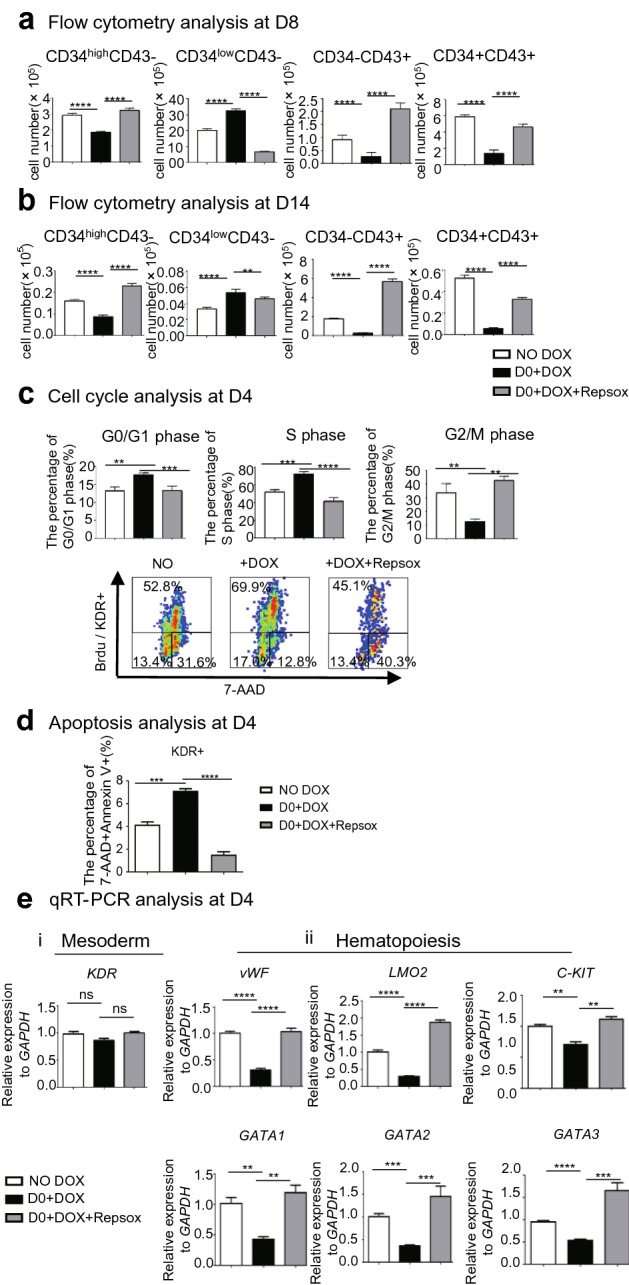
The effects of *P18* overexpression from the early stage on hematopoietic differentiation can be counteracted by inhibition of TGF-β signaling. When *P18*/hESCs co-cultured with AGM-S3 cells were treated with DOX from D0, flow cytometry with 7-AAD and combination of anti-CD34/CD43 antibodies at (**a**) D8 or (**b**) D14 revealed that production of CD34^high^CD43−, CD34−CD43+, and CD34+CD43+ populations was reduced. (**c**) Cell cycle analysis at D4 indicated that the proportion of KDR+ cells in G2/M phase decreased significantly, whereas the proportions of cells in G0/G1 and S phases increased significantly. (**d**) *P18*/hESC cocultured with AGM-S3 cells were treated with or without DOX, or with both DOX and 0.33 μM RepSox started from D0. At D4 these cocultures were performed apoptosis analysis by corresponding kit using 7-AAD and anti-KDR antibody. The results indicated that *P18* overexpression from D0 increased the apoptosis of co-cultures at D4, which can be counteracted by the inhibition of TGF-β signaling. (**e**) Co-cultured *P18*/hESCs were treated without or with DOX or with both DOX and 0.33 μM RepSox from D0, and analyzed by qRT-PCR at D4. The expression of *KDR*, which is related to mesoderm induction, was stable (i), while important hematopoiesis related genes were downregulated (ii). All of these effects can be counteracted by addition of 0.33 μM RepSox from D0. All results were expressed as means ± SD of three repeated experiments, and p < 0.05 was considered significant (*p < 0.05, **p < 0.01, ***p < 0.001, ****p < 0.0001).

*P18*/hESCs co-cultured with AGM-S3 cells were treated with DOX from D0 and subjected to flow cytometry at D4, D8, or D14. The results revealed that production of the CD34^high^CD43−, CD34−CD43+, and CD34+CD43+ populations were reduced at D8 and D14 (Fig. 4a,b, Fig. S2a,b), as confirmed by colony formation assays (Fig. 7a). Cell-cycle analysis of D4 co-cultures revealed that the proportion of KDR+ cells in G2/M phase decreased significantly, whereas the proportions of G0/G1 and S phase cells increased significantly (Fig. 4c). Apoptosis analysis of D4 co-cultures revealed that the proportion of KDR+ cells in apoptosis increased significantly (Fig. 4d, Fig. S5). qRT-PCR analysis at D4 demonstrated that expression of KDR was stable while important hematopoiesis related genes were downregulated with DOX induction (Fig. 4e). Treatment with both DOX and 0.33 μM RepSox counteracted all of these effects (Fig. 4). Thus, the inhibitory effects of *P18* overexpression on the early stage of hematopoiesis might involve the TGF-β signaling pathway and alteration of cell-cycle status and transcription profile of key hematopoietic genes.

### Overexpression of P18 at the late stage promotes hematopoietic differentiation

**Figure 5:**
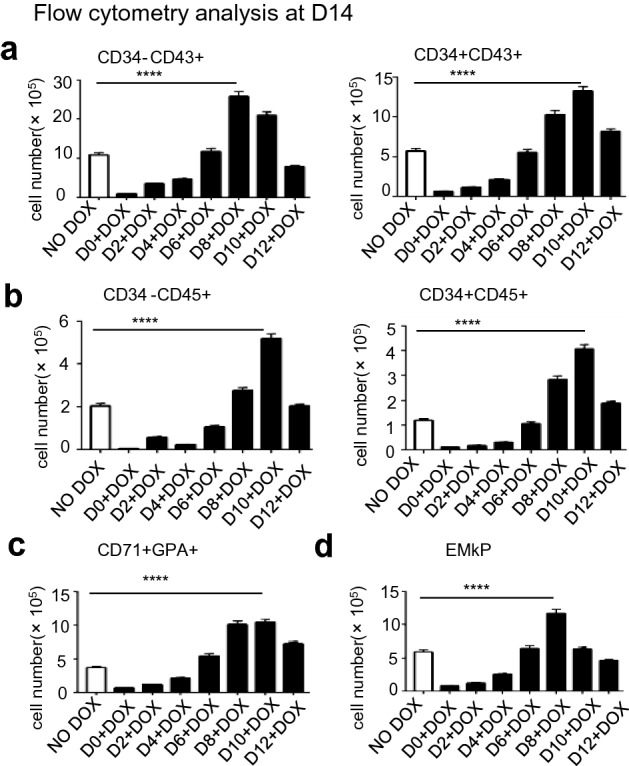
Overexpression of *P18* from D10 promotes hematopoietic differentiation. *P18*/hESCs were co-cultured with AGM-S3, and *P18* was induced from D0, D2, D4, D6, D8, D10, or D12. Flow cytometry at D14 with 7-AAD and the antibodies against (**a**) CD34/CD43, (**b**) CD34/CD45, (**c**) CD71/GPA, (**d**) CD34/CD43/GPA/CD41a (For EMkP) revealed that most of the aforementioned cell populations were dramatically expanded by induction of *P18* from D10, especially the CD34−CD43+, CD34−CD45+, GPA+CD71+, and EMkP populations, indicating that *P18* overexpression strongly promoted hematopoietic differentiation at the late stage. All results were expressed as means ± SD of three repeated experiments, and p < 0.05 was considered significant (*p < 0.05, **p < 0.01, ***p < 0.001, ****p < 0.0001).

*P18*/hESCs co-cultured with AGM-S3 cells treated with DOX from D6 or later, especially from D10, significantly promoted hematopoietic differentiation at the late stage (Fig. 5, Fig. S3). Flow cytometry at D14 revealed that the populations of CD34+CD43+, CD34−CD43+, CD34−CD45+, CD34+CD45+, GPA+CD71+, and erythroid-megakaryocytic progenitor (EMkP)-like cells were dramatically increased by induction of *P18* from D10, indicating that *P18* overexpression strongly promotes hematopoietic differentiation at the late stage.

### P18 overexpression from D10 upregulates NF-κB signaling, promotes hematopoietic differentiation, and changes cell-cycle status and apoptosis, and the most effects can be counteracted by inhibition of NF-κB signaling

**Figure 6:**
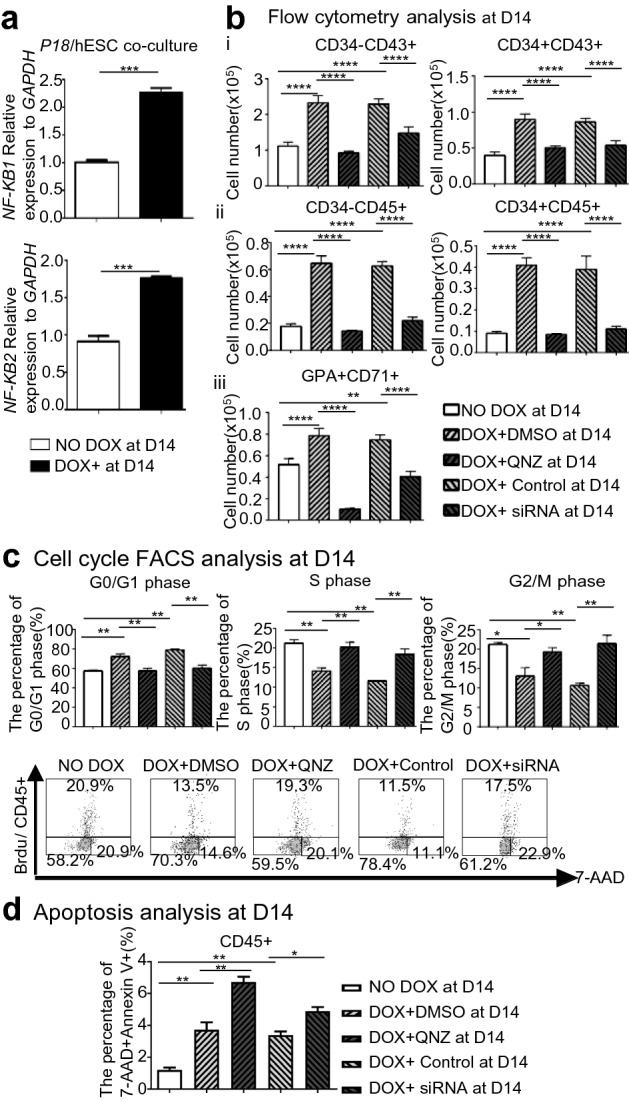
*P18* overexpression from D10 promotes hematopoietic differentiation in a manner that involves NF-κB signaling and might result from alteration of cell cycle statues. (**a**) qRT-PCR detection at D14 revealed that when *P18*/hESCs co-cultured with AGM-S3 cells were treated with DOX from D10, the expression of both *NFKB1* and *NFKB2* was significantly higher than in non-induced cells, indicating that at the later stage, NF-κB signaling was up-regulated by *P18* overexpression. (**b**) *P18*/hESCs co-cultured with AGM-S3 cells were treated from D10 with 20 nM QNZ (an inhibitor of NF-κB signaling) or 20 nM siRNA against *NFKB1*, and flow cytometry at D14 revealed that both treatments attenuated the positive effects of D10-induced *P18* overexpression on the populations of CD34−CD43+, CD34+CD43+, CD34−CD45+, CD34+CD45+, and CD71+GPA+. (**c**) Analyses of cell cycle status revealed that *P18* induction from D10 increased the proportion of CD45+ cells in G0/G1 phase but decreased the proportions of cells in S and G2/M phases; these effects were counteracted by treatment with QNZ or siRNA against *NFKB1*. (**d**) *P18*/hESCs co-cultured with AGM-S3 cells were treated with 20 nM QNZ (or an equal volume of DMSO as control) or 20 nM siRNA against *NFKB1* (or an equal concentration of control siRNA) started from D10. At D14 these cocultures were performed apoptosis analysis by corresponding kit using 7-AAD and anti-CD45 antibody. The results indicated that *P18* overexpression from D10 increased the apoptosis of co-cultures at D14, which can be further promoted with the inhibition of NF-κB signaling. All results were expressed as means ± SD of three repeated experiments, and p < 0.05 was considered significant (*p < 0.05, **p < 0.01, ***p < 0.001, ****p < 0.0001).

When *P18*/hESCs co-cultured with AGM-S3 cells were treated with DOX from D10, qRT-PCR at D14 revealed that expression of both *NFKB1* and *NFKB2* was significantly higher than in non-induced cells (Fig. 6a). Overexpression of *P18* from D10 stimulated hematopoietic populations at D14, including CD34+CD43+, CD34−CD43+, CD34−CD45+, CD34−CD45+, GPA+CD71+ cells. It also increased the proportion of cells in G0/G1 phase, but decreased the proportions of CD45+ cells in S and G2/M phases. Treatment with 20 nM QNZ or 20 nM siRNA against *NFKB1* eliminated this increase (Fig. 6b,c, Fig. S4). Overexpression of *P18* from D10 increased the proportion of CD45+ cells in apoptosis. Treatment with 20 nM QNZ or 20 nM siRNA against *NFKB1* further increased the proportion of CD45+ cells in apoptosis (Fig. 6d, Fig. S6). Together, these observations revealed that *P18* overexpression from D10 promotes hematopoietic differentiation, which is closely related to NF-κB signaling, potentially by altering cell-cycle status.

### Overexpression of P18 blocks colony formation at the early stage and promotes it at the late stage

**Figure 7:**
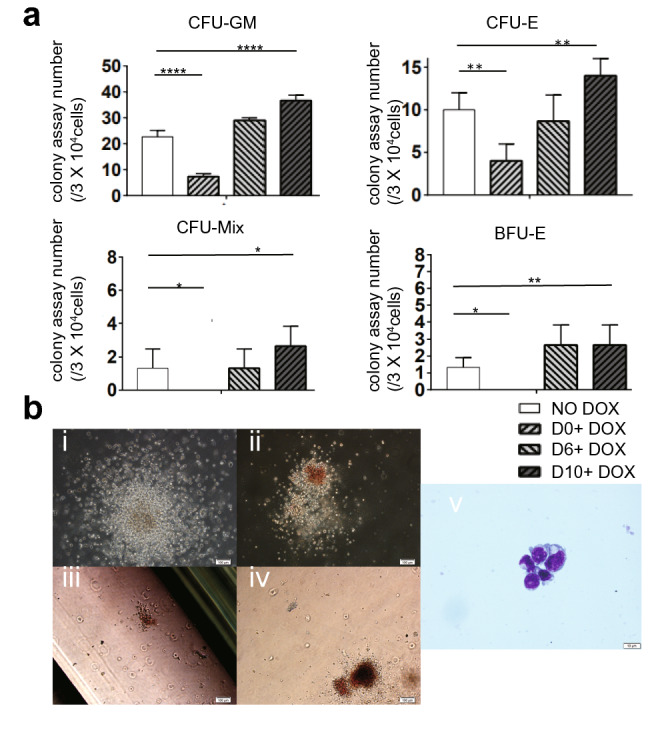
Hematopoietic colony-forming assays. Co-cultured *P18*/hESCs were either not induced or treated with DOX from D0, D6, or D10. At D14, the cells were subjected to an assay to determine their hematopoietic potentials. (**a**) Numbers of colonies derived from 5 × 10^4^ co-cultured cells. p < 0.05 was considered significant. (**b**) Typical morphologies of CFU-GM (i), CFU-Mix (ii), CFU-E (iii), and BFU-E (iv) colonies. Scale bars, 100 µm. MGG staining of cells in BFU-E colonies (v). Scale bar, 10 µm. *CFU-E* colony forming unit–erythrocyte, *BFU-E* burst-forming unit–erythroid, *CFU-GM* colony forming unit–granulocyte/macrophage, *CFU-Mix* colony forming unit–mixture. All results are expressed as means ± SD of three repeated experiments, and p < 0.05 was considered significant. (*p < 0.05, **p < 0.01, ***p < 0.001, ****p < 0.0001).

To further confirm that *P18* overexpression had a negative effect on hematopoiesis, we performed hematopoietic colony assays on co-cultures at D14. *P18* overexpression induced from D0 significantly blocked formation of CFU-GM, CFU-E, CFU-Mix, and BFU-E colonies; however, this inhibitory effect weakened or even disappeared when *P18* was overexpressed from D6, and formation of all colony types was promoted by addition of DOX from D10 (Fig. 7a). The morphologies of typical hematopoietic colonies were examined by phase-contrast microscopy (Fig. 7b i–iv). BFU-E cells were confirmed by May–Grunwald–Giemsa staining (MGG) (Fig. 7b v).

## Discussion

*P18* (*CDKN2C*) is a key CKI during the cell cycle. Knockout, knockdown or chemical inhibition of *P18* in mouse models promotes the generation of HSCs^17,20,21^. To date, however, the effects of up-regulation of *P18* on hematopoiesis have not been deeply explored. Previously, we reported that *RUNX1b* overexpression at the early stage blocks human hematopoiesis^1^. Here, we observed that in *RUNX1b*/hESC co-cultures, expression of *P18* at D4 was upregulated when *RUNX1b* was induced from D0, and that this effect could be counteracted by addition of the TGF-β signaling inhibitor RepSox from D0 (Fig. 1b,c), which indicated that *P18* might be relevant to the inhibitory effects of *RUNX1b* on hematopoiesis and the rescue effects of inhibiting TGF-β signaling inhibition, but both of these phenomena require further exploration. In this study, we have established an inducible *P18*/hESC line to investigate in detail the function of *P18* overexpression (Fig. 1d–h).

The primary results showed us some key clues. At the early stage *P18* overexpression can block the early hematopoietic differentiation, which is similar to the effects of *RUNX1b/c* that can be partially rescued by inhibition of TGF-β signaling^1^. At the late stage it can broadly promote the hematopoietic differentiation, which is similar to the effects of *HOXA9* and *HOXC4*^23,24^ that can be counteracted by inhibition of NF-κB signal pathway, and enhance the expression level of key genes of this pathway. Therefore, we speculated that *P18* function ought to be closely related to TGF-β and NF-κB signaling at the early and late stage of hematopoiesis respectively according to our previous researches and performed further exploration based it.

In the cocultures with hESC and AGM-S3 at D8, CD34+CD43− cells can be produced and obviously divided into two sub-populations: CD34^high^CD43− and CD34^low^CD43− cells. Compared with CD34^low^CD43− cells, CD34^high^CD43− cells were able to produce more CD34+CD43+ cells expression level of CD34 protein, which indicated they might have different traits during hematopoietic differentiation (Fig. 2a). The CD34^high^CD43− cells might be the target population of *P18*, and that overexpression of *P18* at the early stage (especially from D0) can severely decrease the production of these cells and their derivative populations, such as CD34+CD43+ and CD34−CD43+ cells at D8, which effect weakened when it was initiated after D2 and disappeared when it was initiated after D6. The CD34+CD43+ and CD34−CD43+ populations exhibited similar patterns (Fig. 2b). By contrast, the production of CD34^low^CD43− cells was not significantly influenced. In addition, we found that the production of all of the aforementioned populations were influenced in a similar degree between the ones induced from D0 to D2 or from D0 to D8 (Fig. 2c). Overexpression of *P18* from D0 to D2 was sufficient to prevent KDR+ cells in D2 cocultures from producing CD34^high^ cells, but had no obvious influence on the production of CD34^low^ cells (Fig. 3). When induction of *P18* started from D4, the blockage disappeared. Above observation strongly indicated that the blockage of transition from the CD34^low^ to CD34^high^ sub-population happened mostly at the earliest stage of mesoderm induction (D0–D2) and not later than D4. Together, *P18* overexpression in mesodermal populations (mainly KDR+ cells) at the early stage inhibited formation of the CD34^high^ sub-population.

In a previous study, we showed that inhibition of TGF-β signaling partially rescued inhibition of hematopoietic differentiation by *RUNX1b*^1^, and the function of *P18* at the early stage involves TGF-β signaling, a point that warranted investigation. The inhibition of TGF-β signaling could counteract most effects of *P18* overexpression from D0 (Fig. 4a,b). The qRT-PCR analysis of hematopoiesis or mesoderm-related genes indicated that *P18* overexpression probably blocked the mesoderm–hemogenesis transition while not significantly influenced the induction of mesoderm (Fig. 4d). For all these effects could be counteracted by inhibition of TGF-β signaling it is possible that together with TGF-β signaling, *P18* overexpression from D0 significantly altered cell-cycle status, blocking the transition from CD34^low^ to CD34^high^ cells. We inclined to think that with D0–DOX treatment the D4 coculture cells were arrested in G1 stage and their proliferation was significantly retarded, which lead to severe blockage of the transition from CD34^low^CD43− cells to CD34^high^CD43− cells and later significant less production of HSPCs than the control one. It ought to be the main reason that their D14 cocultures produced less colonies compared to the ones with D6-DOX treatment or untreated control ones. The *P18* overexpression started after D6 had not such blockage effects, and the emerge of HSPC started at around D10 in AGM coculture system, at which stage *P18* overexpression had a promote effects on hematopoiesis. Therefore, the cell cycle status of HSPCs (if in quiescent) might help to these prohibitory effects, but probably not be the main reason.

It is surprising that according to the results of flow cytometry the induction of *P18* from D6 or later significantly promoted the production of the aforementioned populations at D8 (Fig. 2a), and that induction of *P18* from D10 significantly promoted the production of classic hematopoietic populations at D14 (Fig. 5). Colony formation assays also confirmed such tendency of hematopoietic potentials changed by *P18* overexpression at the late stages in functional level, consistent with the results of flow cytometry (Fig. 7). This observation indicated that from the middle to the late stage of hematopoiesis, *P18* overexpression broadly promotes hematopoiesis, including myelogenesis and erythrogenesis. *NFKB1* and *NFKB2*, were upregulated when *P18* was induced overexpressed from D10 (Fig. 6a). When NF-κB signaling was inhibited by its inhibitor (QNZ) or by siRNA against *NFKB1*, except for further increased apoptosis, all other effects of *P18* overexpression at the late stage were counteracted (Fig. 6b,c), which was very similar to the phenomenon caused by *HOXC4* and *HOXA9* overexpression at the late stage^23,24^, and indicated that these genes probably share common mechanisms to promote hematopoiesis with the aid of NF-κB signaling.

Xenotransplantation studies should be very helpful to elucidate the more detail function of *p18* on hematopoiesis and show the changes of their potentials of hematopoietic differentiation and lineage bias caused by *P18* overexpression and the inhibition of TGF-β or NF-κB signaling at different stages though we have no enough technique conditions to perform it. Nevertheless, the proofs from flow cytometry and colony formation assays ought to be enough to discover these phenomena, among which the promotion effects of *P18* overexpression on hematopoiesis has been rare reported in a functional study of CKIs.

Because few previous studies had investigated the effects of *P18* overexpression, we carefully monitored its effects on different stages of hematopoietic differentiation and sought to identify the relevant signal pathways. The negative effects at the early stage and the positive effects at the late stage both involved in changes in cell cycle status. Overexpression of *P18* significantly decreased the proportion of D4 KDR+ cells or D14 CD45+ cells in G2/M and increased the proportions of these cells in G0/G1; these effects can be counteracted by the inhibition of TGF-β or NF-κB signaling. To the cells in S stage, the changes of cell cycle status caused by *P18* overexpression was different at the different stages, which were also counteracted by the inhibition of the corresponding signaling (Figs. 4c, 6c). This indicated that both signaling pathways are involved in the effects of *P18* overexpression on cell cycle status, with distinct consequences in different stages and contexts.

From the view of apoptosis, the *P18* overexpression at the early or late stage can both promote the apoptosis. Inhibition of TGF-β signaling can counteract it at early stage while NF-κB signaling can promote it at the late stage (Figs. 4d, 6d). It is very interesting that the members of INK4 family was only reported to negatively regulate apoptosis^26^. Some INK4 member, such as *P16*, was inactive by hypermethylation and homozygous deletions in leukemia-lymphoma cell lines, and ectopic expression of *P16* in such cell line will lead to growth inhibition, arrest in G1 without apoptosis and rare differentiation^27^. It is very similar to the effects of *P18* in hematopoiesis at the early stage except that *P18* overexpression can increase the apoptosis, which was also closely related to TGF-β signaling. But in renal tubular epithelial cells (LLC-PK1) *p18* overexpression reduced the percentage of apoptotic cells significantly^28^, which indicated that it effects on apoptosis seem distinctive in different context and cell type.

**Figure 8:**
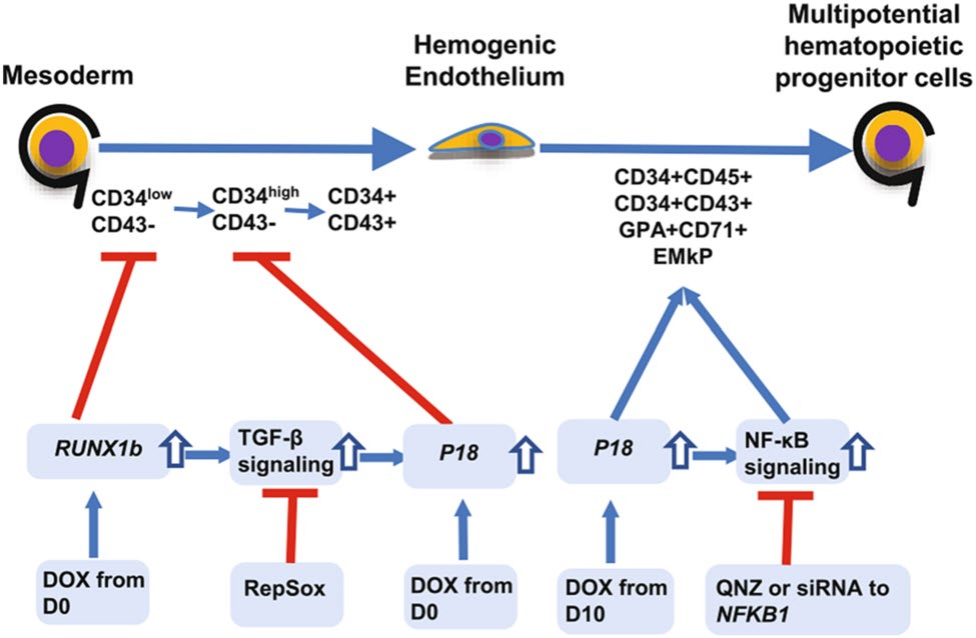
The diagram of control mechanisms for the functions of *P18* on hematopoiesis. The *RUNX1b* overexpression can upregulate TGF-β signaling and *P18*, and the inhibition of TGF-β signaling can restore the expression level of *P18*. The *P18* overexpression at D0 can severely decrease the product of CD34^high^CD43− cells and their derived populations but not CD34^low^CD43− cells that can only be blocked by *RUNX1b* overexpression at D0. The *P18* overexpression at D10 can significantly increase the product of classic hematopoietic populations, which can be counteracted by the inhibition of NF-κB signaling. *P18* has distinctive function on hematopoietic differentiation at different stage, which are both related to the change of cell cycle status.

*p18* overexpression from D0 can block transition from CD34^low^CD43− cells to CD34^high^CD43− cells so as to prevent the emerging of hematopoietic endothelium and their derived hematopoietic stem/progenitor cells. *p18* overexpression from D10 can promote the development of hematopoietic stem/progenitor cells and other progenitors of classic hematopoietic lineage. It is reasonable to speculate that both of them ought to be caused by similar effects that G1 arrest, proliferation blockage and apoptosis were increased, but they influence the production of hematopoietic stem/progenitor cells with contrary style, which detail mechanism need further exploration. The primary mechanisms and functions of *p18* overexpression in hematopoiesis were summarized in Fig. 8.

At the early stage of AGM-S3 co-culture, overexpression of *P18* and *P21* had opposing effects on the proportions of cells in S and G2/M, but similar inhibitory effects on hematopoietic differentiation. All of these effects could be counteracted by inhibition of TGF-β signaling, as in the case of *RUNX1b*^1,29^. Overexpression of any of these genes increased the proportion of cells in G0/G1, indicating that TGF-β signaling plays a key role in G1 arrest. Stimulation of TGF-β signaling mediates cell-cycle arrest and up-regulates expression of *P15*^30–32^*.*In our co-culture system, *RUNX1b* overexpression stimulated TGF-β signaling, which upregulates not only *P15* (unpublished data) but also *P18* and *P21*. *P18*, *P15* and other members of the INK4 family contain repeated ankyrin motifs and can regulate G1 phase by inhibiting CDK4/6 and interfering with cyclin–CDK assembly, thereby inducing G1 arrest^15,33,34^. *P21*, which belongs to the Cip/Kip family, does not contain repeated ankyrin motifs and therefore cannot specifically bind to CDK4 and CDK6; consequently, its mechanism is distinct from that of *P18*^19^; however, the two proteins appear to have similar effects on hematopoiesis at the early stage. It is reasonable to speculate that with the help of TGF-β signaling, *RUNX1* might coordinate and organize various CKIs (such as *P15*, *P18*, and *P21*) to control early hematopoietic differentiation via distinct mechanisms.

In contrast to *P21*, *P18* overexpression at the late stage efficiently promotes hematopoietic differentiation, potentially due to differences in protein structure^15,34^. Similar effects can be observed following overexpression of *HOXA9* and *HOXC4* at the same stage^23,24^. Overexpression of these factors changed the cell cycle status with different styles while the inhibition of NF-κB signaling can counteract all these changes except for apoptosis, which reveal it involved in the function of *P18* on hematopoietic differentiation through the controlling of cell cycle status but not apoptosis. This study provides the first evidence that *P18* overexpression promotes hematopoiesis, a property that is very rare among CKIs. The underlying mechanism, which requires further exploration, may have important clinical applications.

## Supplementary Information

Supplementary Information 1.

Supplementary Information 2.

## Data Availability

All date generated or analyzed during this study are included in the published article and its supplementary information files.
