# Supplementary material for: The distinct effects of P18 overexpression on different stages of hematopoiesis involve TGF-β and NF-κB signaling
Source: Sci Rep. 2021 Dec 14;11:24014. doi: 10.1038/s41598-021-03263-2 (PMC8671498; doi:10.1038/s41598-021-03263-2)

Supplemental Information

Supplemental materials and methods

Construction of P18 (CDKN2C) inducible hESC lines

The coding sequence (CDS) of *P18* (NM_001262.2) was synthesized by Qingke Inc (Beijing, China) and inserted into the PB-tet-on-OE vector to yield the *P18* inducible vector PB-tet-on-GFP-T2A-CDKN2C. H1 hESCs (generously provided by Prof. Cheng Tao) were co-transfected with this vector and the helper plasmid (PB2000PA-1) using the Lipofectamine 3000 Transfection Kit (Invitrogen). Transformants were selected with puromycin (1 μg/ml) and continually passaged using ReleSR (STEM CELL) to establish *P18*-inducible hESC lines. The efficiency and stringency of *P18* overexpression were confirmed by qRT-PCR and western blotting, and the pluripotency of transgenic hESCs treated with or without doxycycline (DOX) was confirmed by western blotting for OCT4, SOX2, and NANOG. qRT-PCR primers and WB antibodies are listed in Tables S1 and S4, respectively.

Quantitative reverse transcription PCR (qRT-PCR) analysis

Total RNA was extracted by TRizol (life technologies) and purified according to the manufacturer's manual. The reverse transcription to complementary DNA (cDNA) was conducted by iScript cDNA Synthesis Kit (bio-rad). The reaction system contained 4 μl 5× Mixture, 1 μl reverse transcriptase, 1 μg total RNA, and nuclease-free water filled to 20 μl. The conditions used for reverse transcription were as follows: 25°C for 5 min, 42°C for 30 min, 85°C for 5 min, and storage at 4°C. qPCR was performed using Fast Start Universal SYBR Green Master (Roche) on a CFX96™ real-time system (Bio-Rad). Each 15 μl reaction contained 7.5 μl 2×Mixture, 0.4 μl each primer (10 μM), 4.7 μl H_2O, and 2 μl complementary DNA. The PCR amplification conditions were as follows: denaturation at 10 min at 95°C, followed by 45 cycles of 95°C for 15 sec, 58°C for 30 sec, and 72°C for 30 sec. Glyceraldehyde 3-phosphate dehydrogenase (*GAPDH*) served as an internal control. Three repeated experiments were set for all reactions, qRT- PCR primers were designed by Primer3 software and listed in Table S1.

Flow cytometry and cell sorting

P18/hESC co-culture cells were treated under different conditions, and classic hematopoietic populations were detected at D8 or D14 by flow cytometry using the corresponding antibody combinations (BD FACS Canto II). After the medium was aspirated, the co-cultures cells were washed twice with Dulbecco's Phosphate Buffered Saline (D-PBS), digested with 0.05–0.25% trypsin-EDTA solution (Invitrogen), centrifuged at 400 g for 5 min, and then resuspended in 200 μl of Sorting Medium (SM, 2% Fetal Bovine Serum in D-PBS). After counting, the cells were filtered through 40-μm nylon meshes, blocked with rabbit serum on ice for 20 min, and then incubated on ice for 30 min with fluorescence-labeled flow antibodies and 7-amino-actinomycin D (7-AAD). After two washes with D-PBS, co-culture cells were subjected to flow cytometry (BD FACS Canto II) or cell sorting on a FACSJazz Sorter (BD Biosciences) at D2 or D6. The data were analyzed using the FlowJo 10 software. The flow antibodies were listed in Tables S2.

Cell cycle analysis

P18/hESC co-cultures were treated with or without DOX, or with DOX and RepSox, from D0. Some samples were treated with 10 μM BrdU for 12 h at D4. Other samples were treated without DOX, or with DOX and QNZ/siRNA together, from D10, and then incubated with 10 μM BrdU for 12 h at D14. The co-cultures were dissociated by treatment with 0.05–0.25% trypsin-EDTA solution, stained with 7-AAD and anti-KDR antibody (D4) or the anti-CD45 antibody (D14), and then subjected to cell-cycle analysis using the APC-BrdU Flow Kit (BD Biosciences). Finally, the cells were subjected to flow cytometry. The sequences of siRNA were listed in Tables S3.

Western blot analysis

Transgenic *P18*/ hESCs induced by DOX or without induction for 48 h, or the D4 *RUNX1b*/hESC co-cultures under different culture condition, were lysed in 500 μl of PMSF-containing RIPA buffer (Sigma). Then the cells were centrifuged at 4 °C at 12,000 g for 15 min, collecting the supernatant. Protein concentration was determined by BCA method. Cell lysates (25 μg each) were denatured at 100 °C for 10 min with 2× loading buffer, and subjected to SDS-PAGE (5% separating gel/10% stacking gel). Polyvinylidene fluoride (PVDF) membrane was used to proteins transfer at 300 mA for 90 min. After that, the membrane was sealed with the blocking solution (1X TBST containing 5% skimmed milk powder) for 1hr, and then incubated with anti-GAPDH (diluted 1:2000, Kangchen Inc.,

Cat#: KC-5G4) or anti-P18 (diluted 1:200, Santa Cruz Biotechnology, Cat#: sc-365,644) mouse monoclonal IgG in blocking solution overnight at 4 °C, and then with horseradish peroxidase-conjugated goat anti-mouse IgG (diluted 1:4000, Abcam, Cat#: ab97040) in blocking solution. Blots were developed using the Amersham ECL Prime western blotting detection kit (GE Healthcare). Use ImageQuant LAS4000 mini system to quantify the signal. The molecular weight was calculated by referring to the pre-stained protein molecular weight reference (PageRuler). Western blot analysis of *P18*/hESC were also confirmed the pluripotency using the antibodies against stemness related markers such as OCT4, SOX2 and NANOG. All the antibodies were listed in Table S4.

Apoptosis analysis

P18/hESC co-cultures were treated with or without DOX, or with both DOX and RepSox from D0, dissociated by treatment with 0.05% trypsin-EDTA solution, stained with 7-AAD and anti-KDR antibody at D4. The same samples were treated with or without DOX, or with DOX and QNZ/NFKB1-siRNA together, or with DOX and DMSO/NC-siRNA together from D10, dissociated by treatment with 0.25% trypsin-EDTA solution, stained with 7-AAD and anti-CD45 antibody at D14. And then subjected to cell apoptosis analysis using the Annexin V-APC/7-AAD Apoptosis Detection Kit (Elabscience Inc, China). 5×10^5 resuspended cells were pellet by 300 × g for 5 min, and resuspended in 100 μL of diluted 1× Annexin V Binding Buffer working solution, and then added 2.5 μL of Annexin V-APC and 2.5 μL of 7-AAD staining solution, gently vortexed to mix well, and incubated at room temperature in dark for 15-20 min. And then the cells were added 400 μL of diluted 1 × Annexin V Binding Buffer and mixed well. Finally, the apoptosis analysis of the cells was subjected to flow cytometry.

Supplementary Figure Legends

Supplementary Figure 1. Overexpression of *P18* from D0 inhibits hematopoiesis in co-culture with AGM-S3 cells *P18*/hESCs were treated with DOX or without DOX starting from D0, D2, D4, D6 or only from D0 to D2, and flow cytometry analysis happened at D8 using 7-AAD and anti-CD34/CD43 antibodies to compare non-induced co-cultures and the GFP+ fractions of co-cultures treated with DOX. Overexpression of *P18* from D0 blocked hematopoietic differentiation in co-cultures with AGM-S3 cells.

Supplementary Figure 2. The effects of *P18* overexpression from the early stage on hematopoietic differentiation can be counteracted by inhibition of TGF-β signaling. When *P18*/hESCs co- cultured with AGM-S3 cells were treated with DOX from D0, flow cytometry analysis at (A) D8 or (B) D14 using 7-AAD and combination of anti-CD34/CD43 antibodies showed that the productions of $CD34^{high}CD43-$, CD34−CD43+, and CD34+CD43+ populations were decreased.

Supplementary Figure 3. Overexpression of *P18* from D10 promoted hematopoietic differentiation. *P18*/hESCs were co-cultured with AGM-S3, and *P18* was induced starting from D0, D2, D4, D6, D8, D10, D12. The flow cytometry analysis at D14 using 7-AAD and the following combinations of antibodies against (A) CD34/CD43, (B) CD34/CD45, (C) CD71/GPA, (D) CD34/CD43/GPA/CD41a (For EMkP).

Supplementary Figure 4. *P18* overexpression from D10 promoted hematopoietic differentiation, which effects were closely related to NF-κB signaling and might be caused by changing of the cell cycle statues. *P18*/hESCs co-cultured with AGM-S3 cells were treated with 20 nM QNZ or 20 nM siRNA against *NFKB1* starting from D10, and flow cytometry analysis atD14.

Supplementary Figure 5. The flow cytometry results of apoptosis analysis in D4 cocultures. *P18*/hESC cocultured with AGM-S3 cells were treated with or without DOX, or with both DOX and 0.33 μM RepSox started from D0. At D4 these cocultures were performed apoptosis analysis by corresponding kit using 7-AAD and anti-KDR antibody. The results indicated that *P18* overexpression from D0 increased the apoptosis of co-cultures at D4, which can be counteracted by the inhibition of TGF-β signaling.

Supplementary Figure 6. The flow cytometry results of apoptosis analysis in D14 cocultures. *P18*/hESCs co-cultured with AGM-S3 cells were treated with 20 nM QNZ (or an equal volume of DMSO as control) or 20 nM siRNA against NFKB1 (or an equal concentration of control siRNA) started from D10. At D14 these cocultures were performed apoptosis analysis by corresponding kit

using 7-AAD and anti-CD45 antibody. The results indicated that *P18* overexpression from D10 increased the apoptosis of co-cultures at D14, which can be further promoted with the inhibition of NF-κB signaling.

Supplementary Tables

Supplementary Table S1. Primers used for qRT-PCR analysis

Gene	Forward primer (5′→3′)	Reverse primer (5′→3′)	Reference ID
CDKN2C	CTCACACGGCTCAAGTCACC	CGCAGTCCTTCCAAATCCATTT	NM_001262.2
NF-KB1	GAAGAGGAAGAAAATGGTGGAGT	AAACACAGAGGCTGGTTTTGTAA	NM_001165412
NF-KB2	GAGAGAAGCCGCAACCAGAG	GGGTTGTAGCAACTCTCCATGTC	NM_001077494
GAPDH	GTCTCCTCTGACTTCAACAGCG	ACCACCCTGTTGCTGTAGCCAA	NM_002046.7
KDR	GGAACCTCACTATCCGCAGAGT	CCAAGTTCGTCTTTTCCTGGGC	NM_002253
GATA1	CACGACACTGTGGCGGAGAAAT	TTCCAGATGCCTTGCGGTTTCG	NM_002049
GATA2	CAGCAAGGCTCGTTCCTGTTCA	ATGAGTGGTCGGTTCTGCCCAT	NM_001145661
GATA3	GCGAGACAGAGCGAGCAA	ACTGGGTACGGCAGAATAAAA	NM_001002295.1
c-KIT	CACCGAAGGAGGCACTTACACA	TGCCATTCACGAGCCTGTCGTA	NM_000222
vWF	CCTTGAATCCCAGTGACCCTGA	GGTTCCGAGATGTCCTCCACAT	NM_000552
LMO2	GCGCCTCTACTACAAACTGGGC	CTCATAGGCACGAATCCGCTTG	NM_001142315

Supplementary Table S2. Antibodies used in flow cytometric analysis

Antigen	Brand	Antibody channels	Clone	Cat
CD309(KDR)	Biolegend	PE	7D4-6	359904
CD34	BD	APC	581	555824
CD43	BD	PE	1G10	560199
CD45	BD	PE-CY7	HI30	557748
CD235a(GPA)	BD	APC	GA-R2 (HIR2)	551336
CD71	BD	PE	M-A712	555537
CD38	BD	PerCP-CY5	HIT2	551400
CD90	BD	PE-CY^{TM}7	5E10	561558
7-AAD	BD	7-AAD		559925

Supplementary Table S3. siRNA sequence of *NFkB1*.

Gene name	sense (5′→3′)	antisense (5′→3′)
NFKB1-1	CGAAUGACAGAGGCGUGUAUATT	UAUACACGCCUCUGUCAUUCGTT
NFKB1-3	CCUUUCCUCUACUAUCCUGAATT	UUCAGGAUAGUAGAGGAAAGGTT

Supplementary Table S4. Antibodies for WB

Antigen	Source	Specificity	Cat
CDKN2C	Santa Cruz	Mouse to Human	sc-9965
NANOG	Santa Cruz	Mouse to Human	sc-293121
SOX2	Santa Cruz	Mouse to Human	sc-365823
OCT4	Santa Cruz	Mouse to Human	sc-5279
GAPDH	KANG CHEN	Mouse to Human	KC-5G4
Mouse IgG(H+L)	ZSBIO	Goat to Mouse	ZB-2305

Figure S1

a Flow cytometry analysis at D8

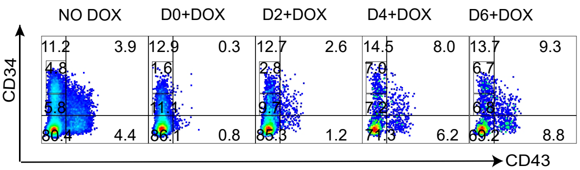

b Flow cytometry analysis at D8

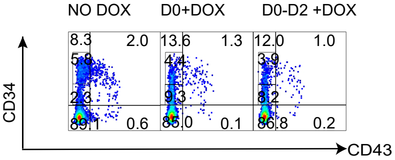

Figure S2

a Flow cytometry analysis at D8

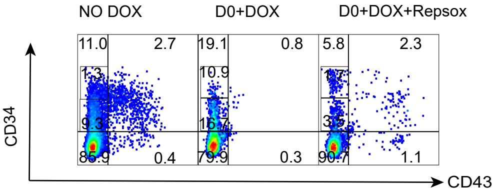

b Flow cytometry analysis at D14

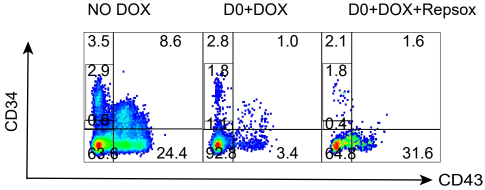

Figure S3

Flow cytometry analysis at D14

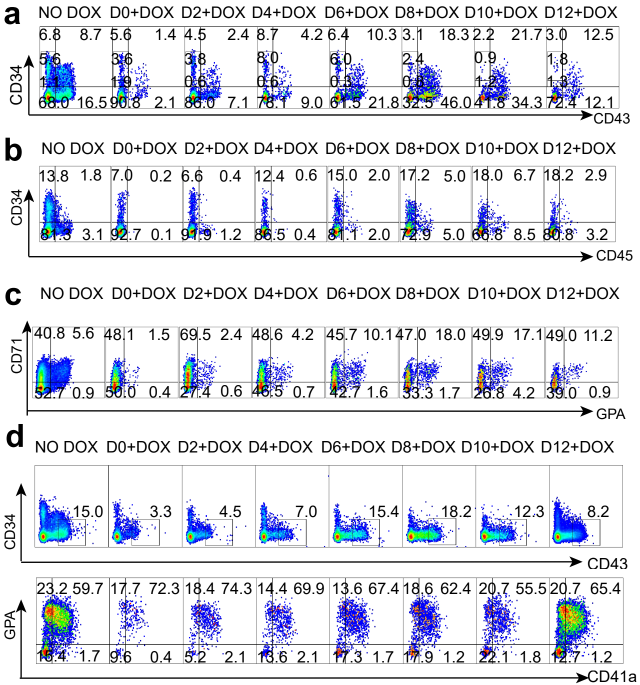

Figure S4

Flow cytometry analysis at D14

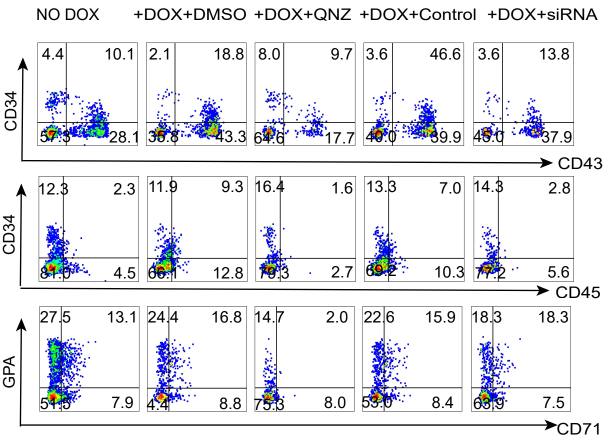

Figure S5

Apoptosis analysis at D4

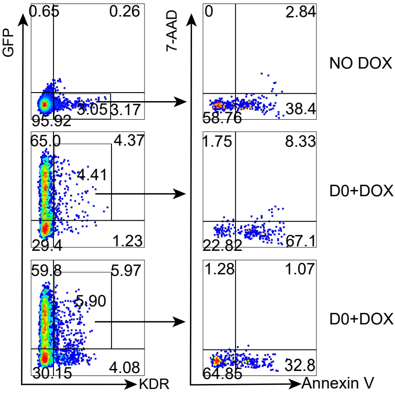

Figure S6

Apoptosis analysis at D14

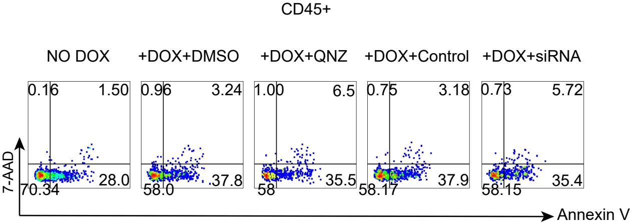

Supplement: Supplementary file 1 — Supplementary Information 1. [file 41598_2021_3263_MOESM1_ESM.pdf]
