# Supplementary material for: The distinct effects of P18 overexpression on different stages of hematopoiesis involve TGF-β and NF-κB signaling
Source: Sci Rep. 2021 Dec 14;11:24014. doi: 10.1038/s41598-021-03263-2 (PMC8671498; doi:10.1038/s41598-021-03263-2)
Supplement: Supplementary file 2 — Supplementary Information 2. [file 41598_2021_3263_MOESM2_ESM.pdf]

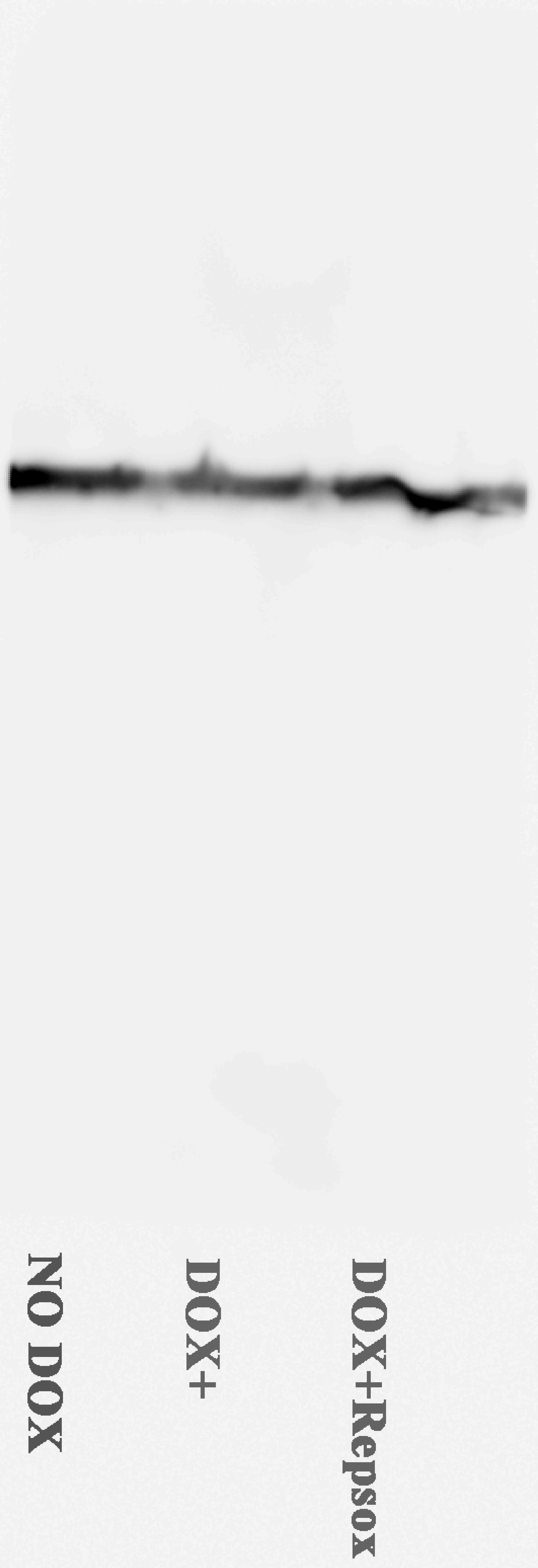

Fig 1C_GAPDH_1

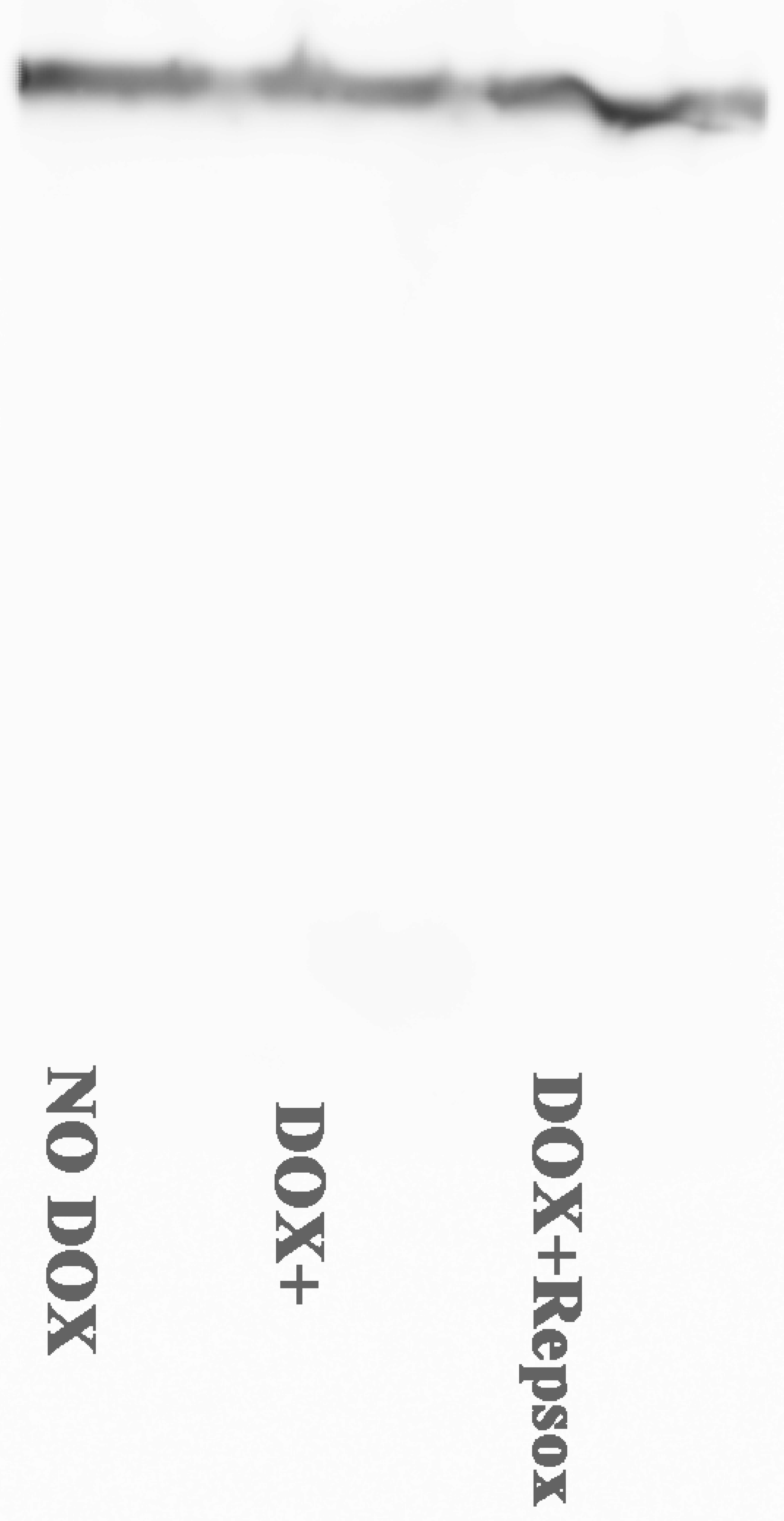

Fig 1C_GAPDH_2

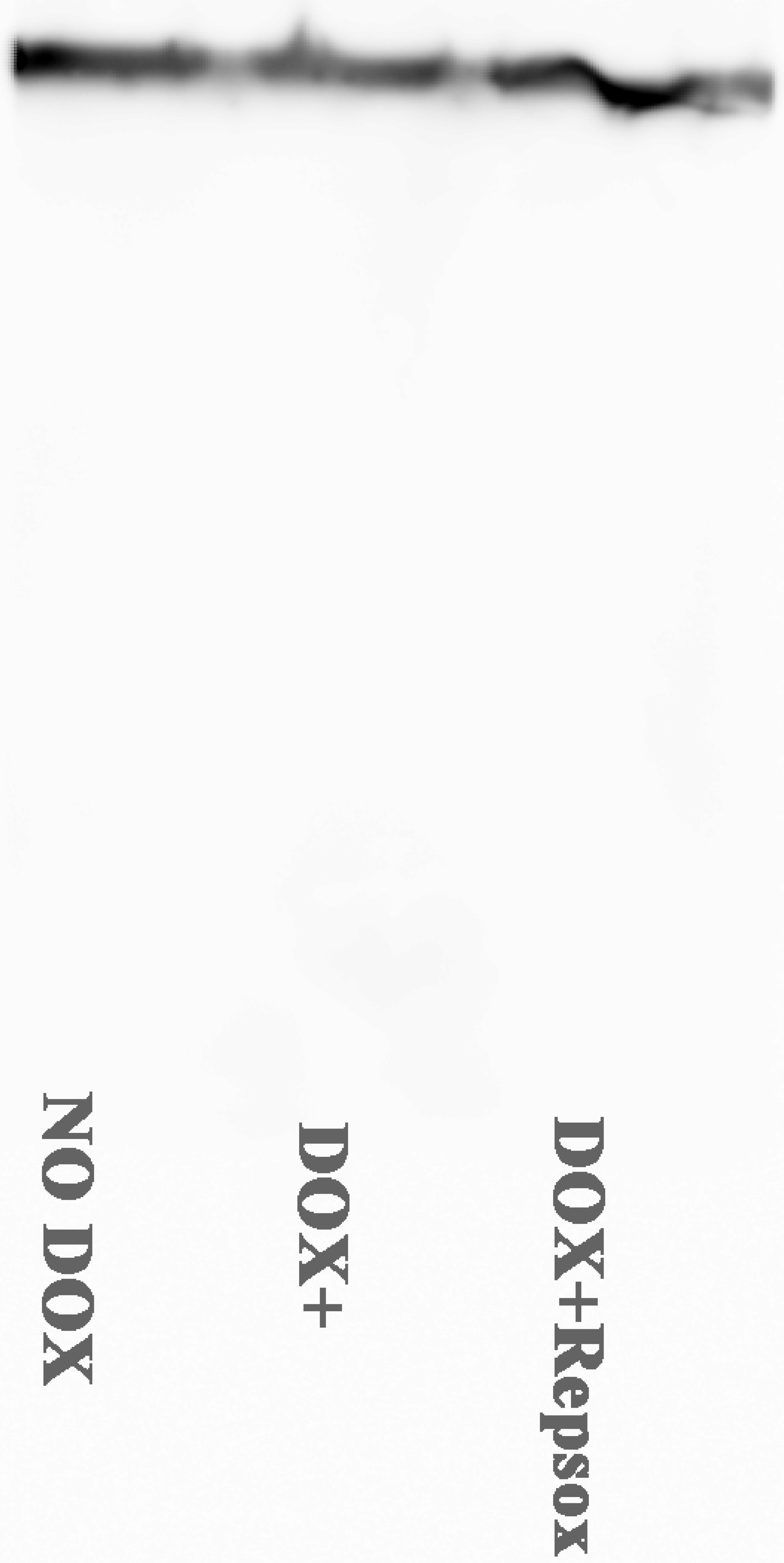

Fig 1C_GAPDH_3

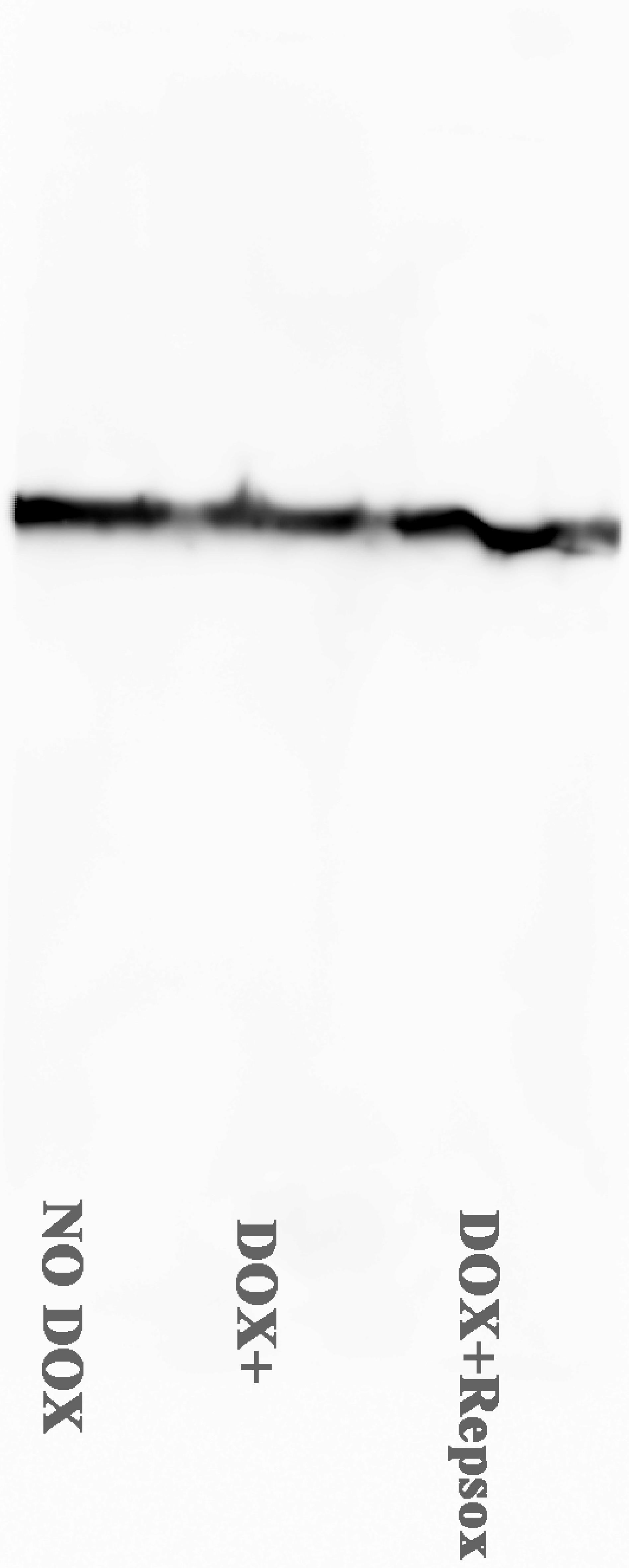

Fig 1C_GAPDH_4

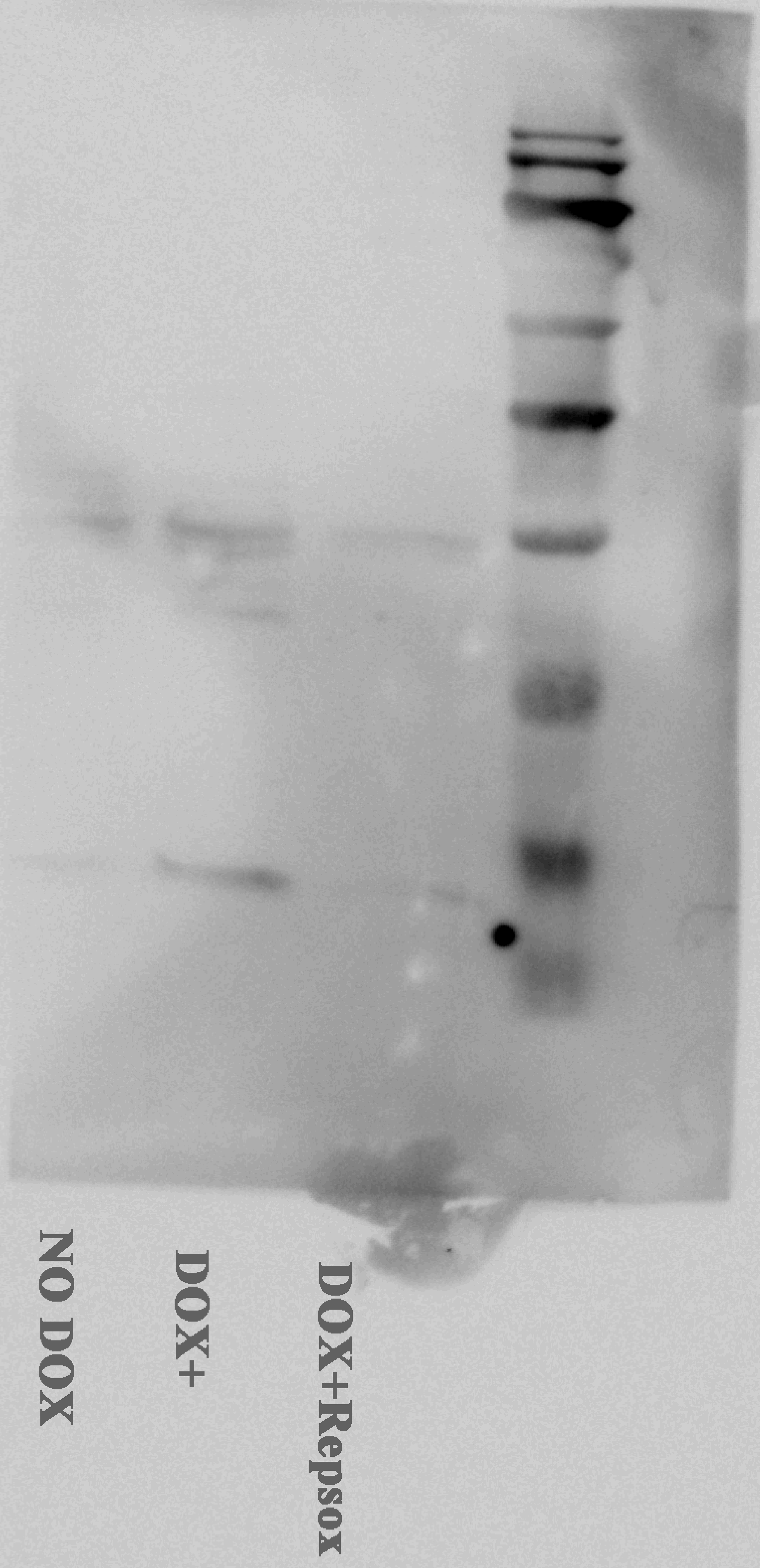

Fig 1C_P18_1

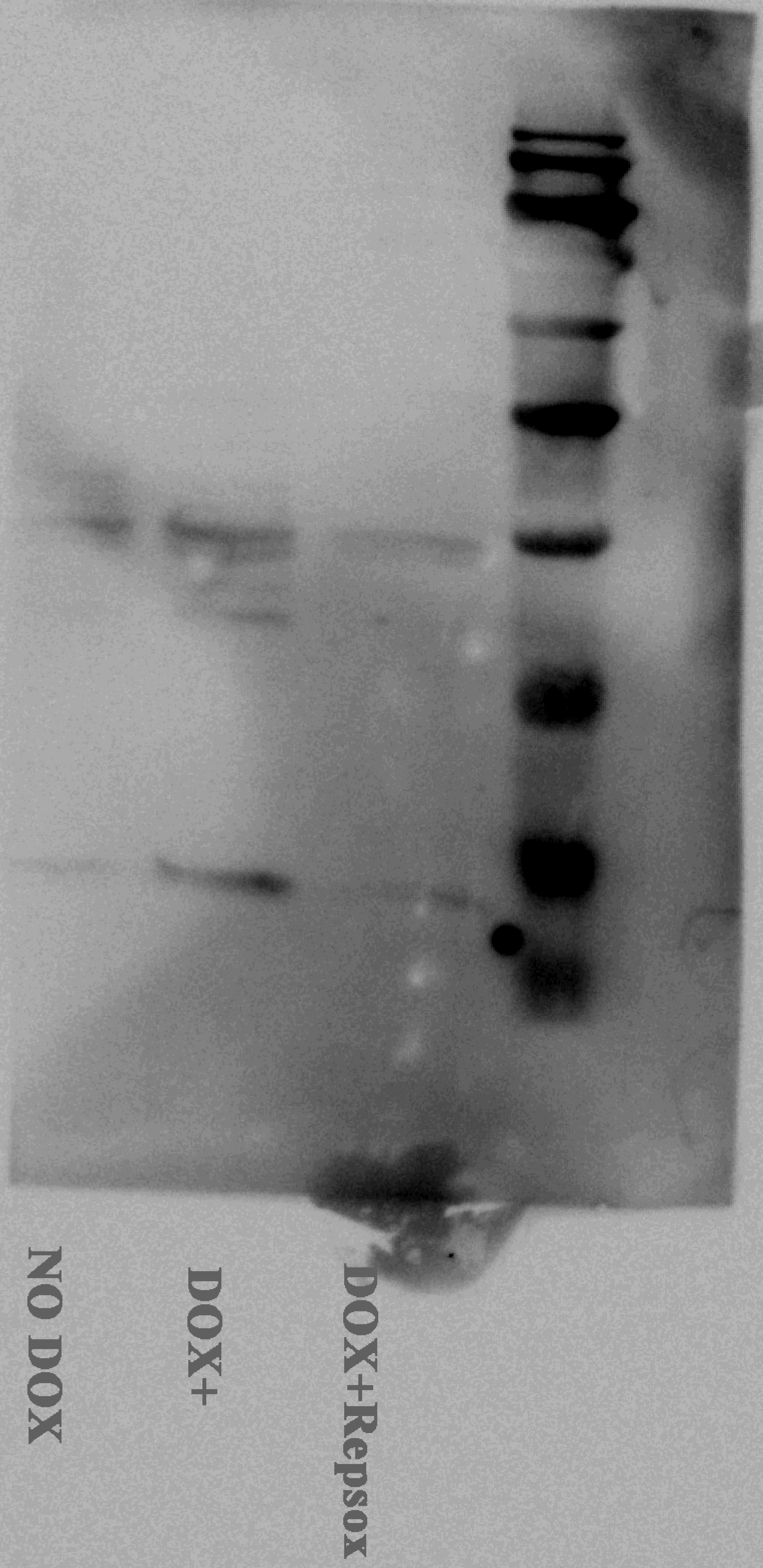

Fig 1C_P18_2

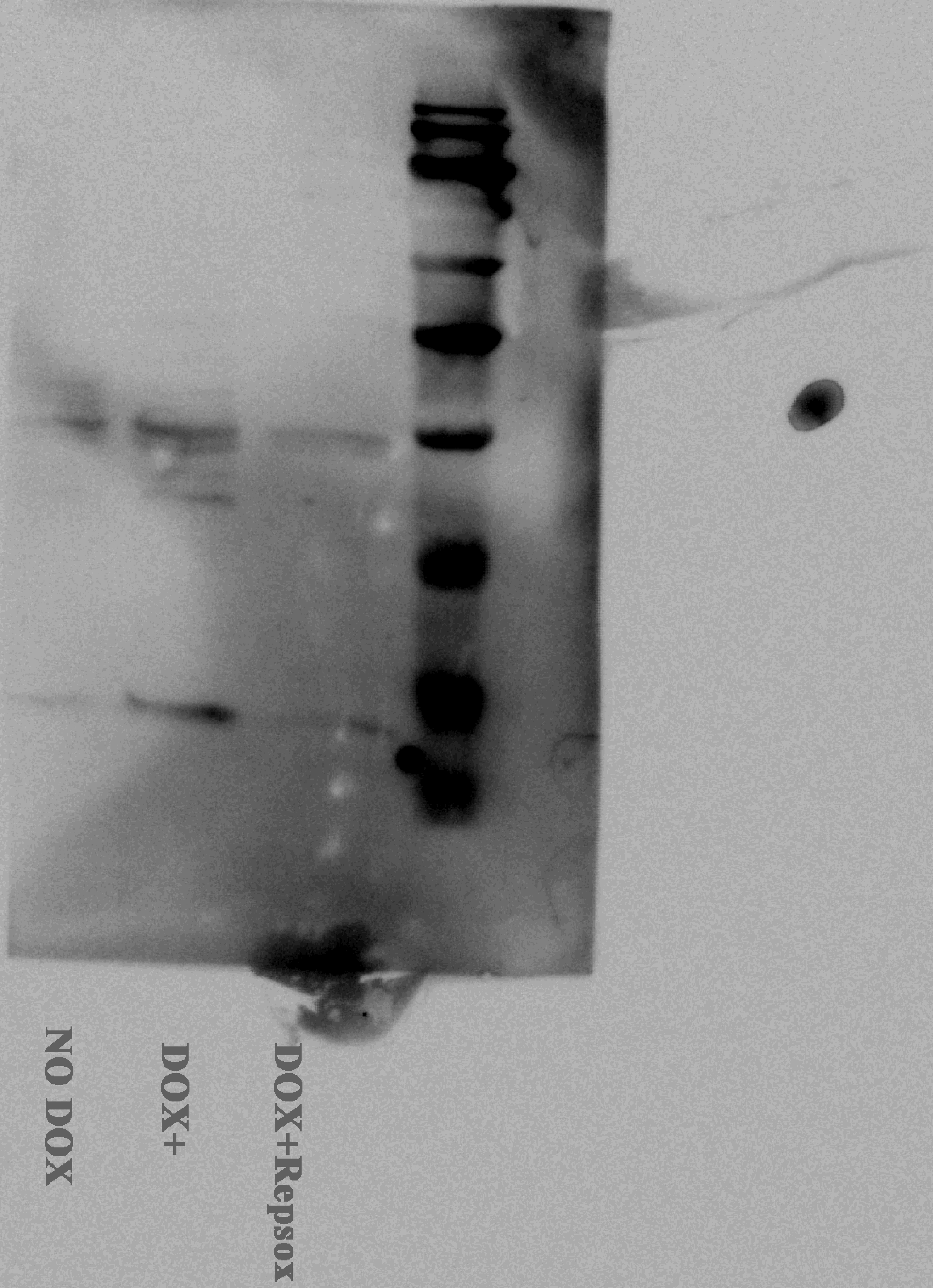

Fig 1C_P18_3

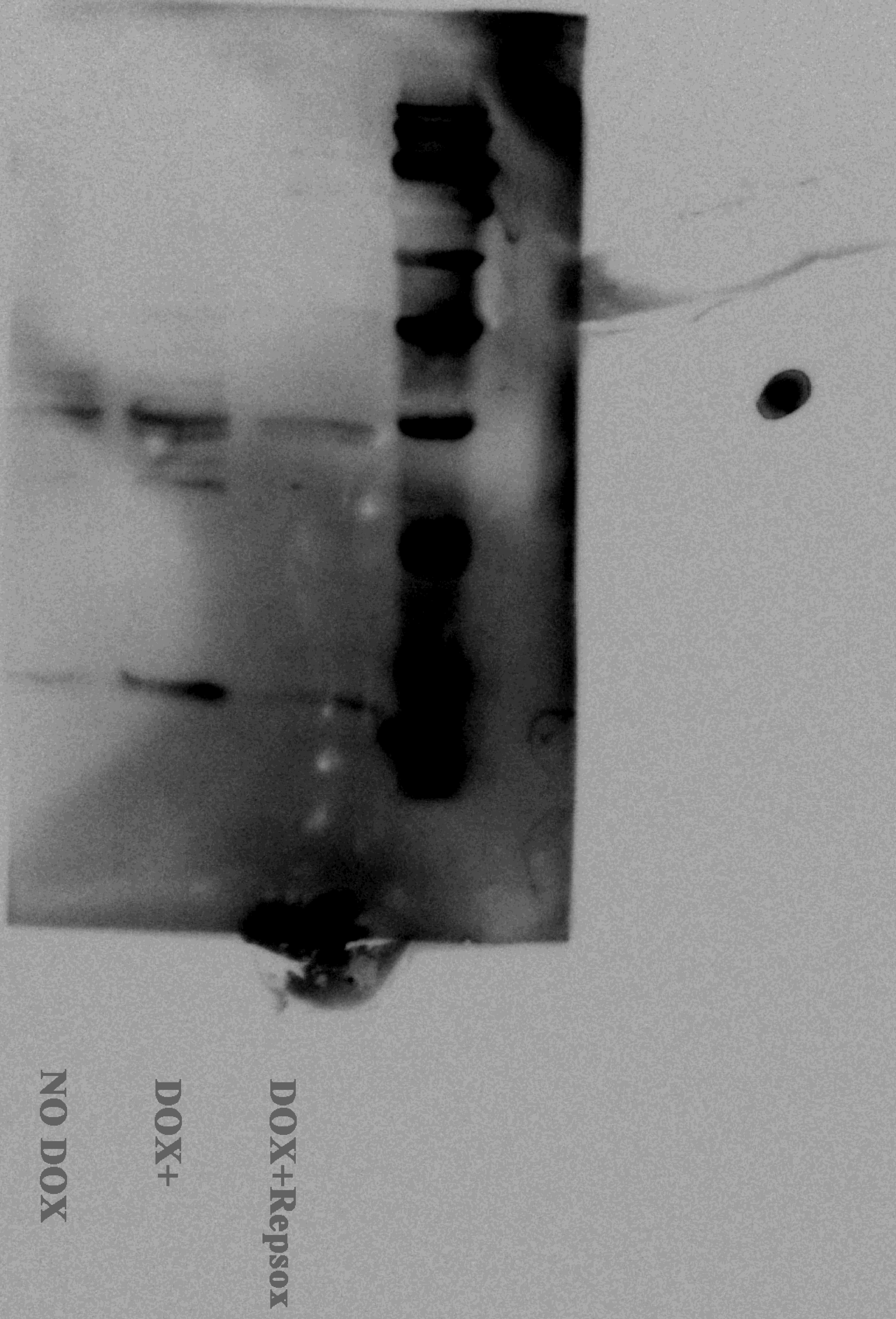

Fig 1C_P18_4

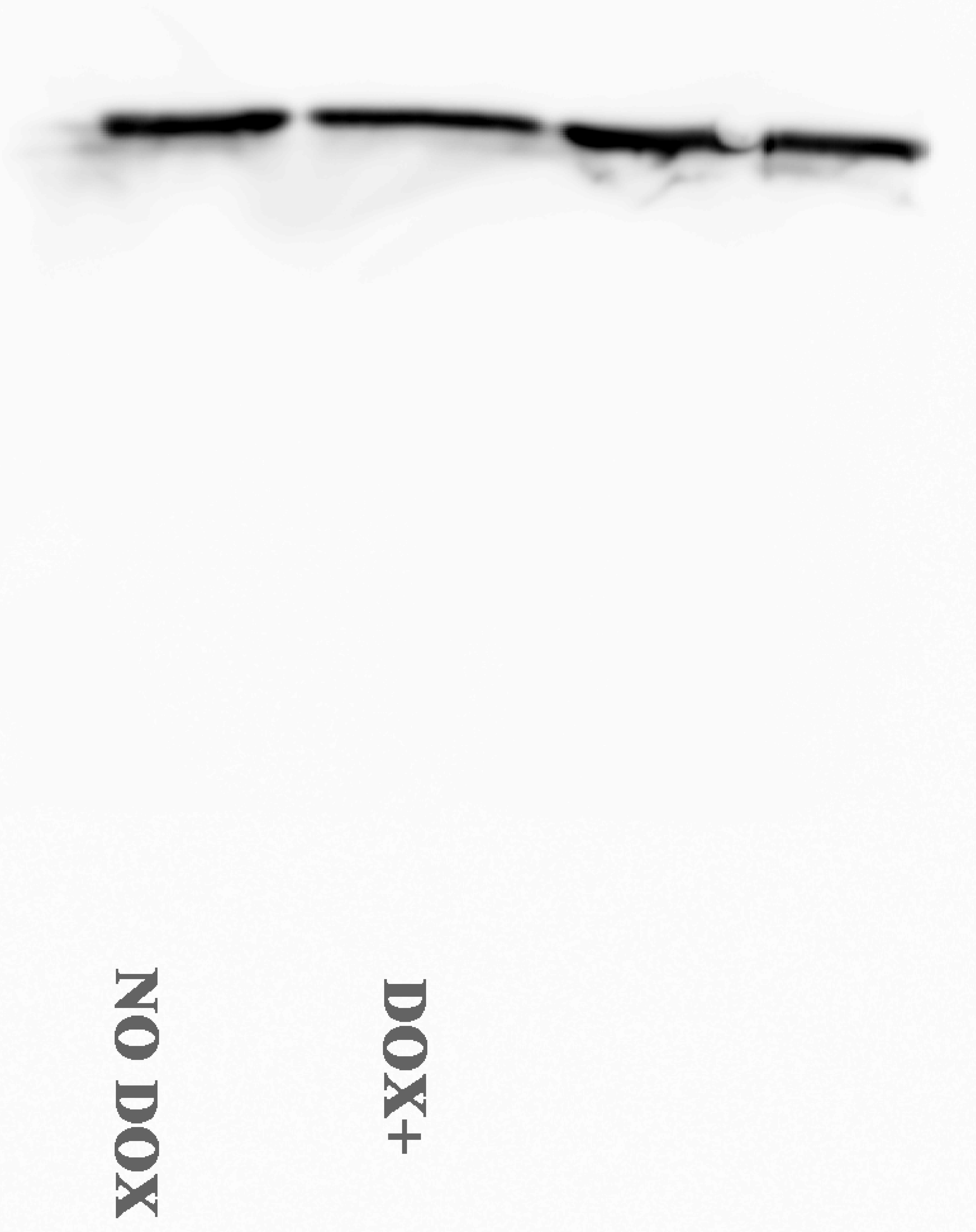

Fig 1G_GAPDH_2

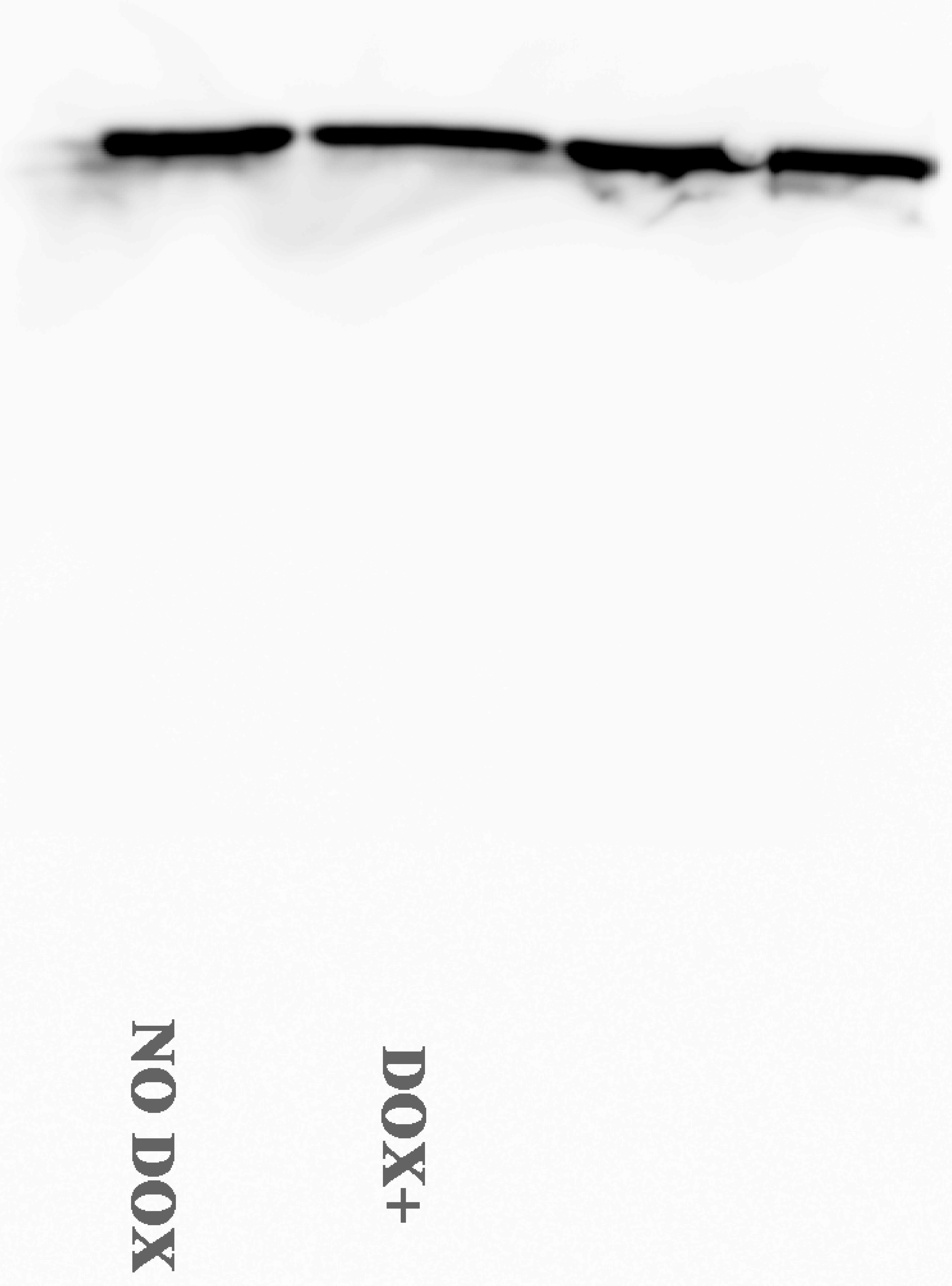

Fig 1G_GAPDH_3

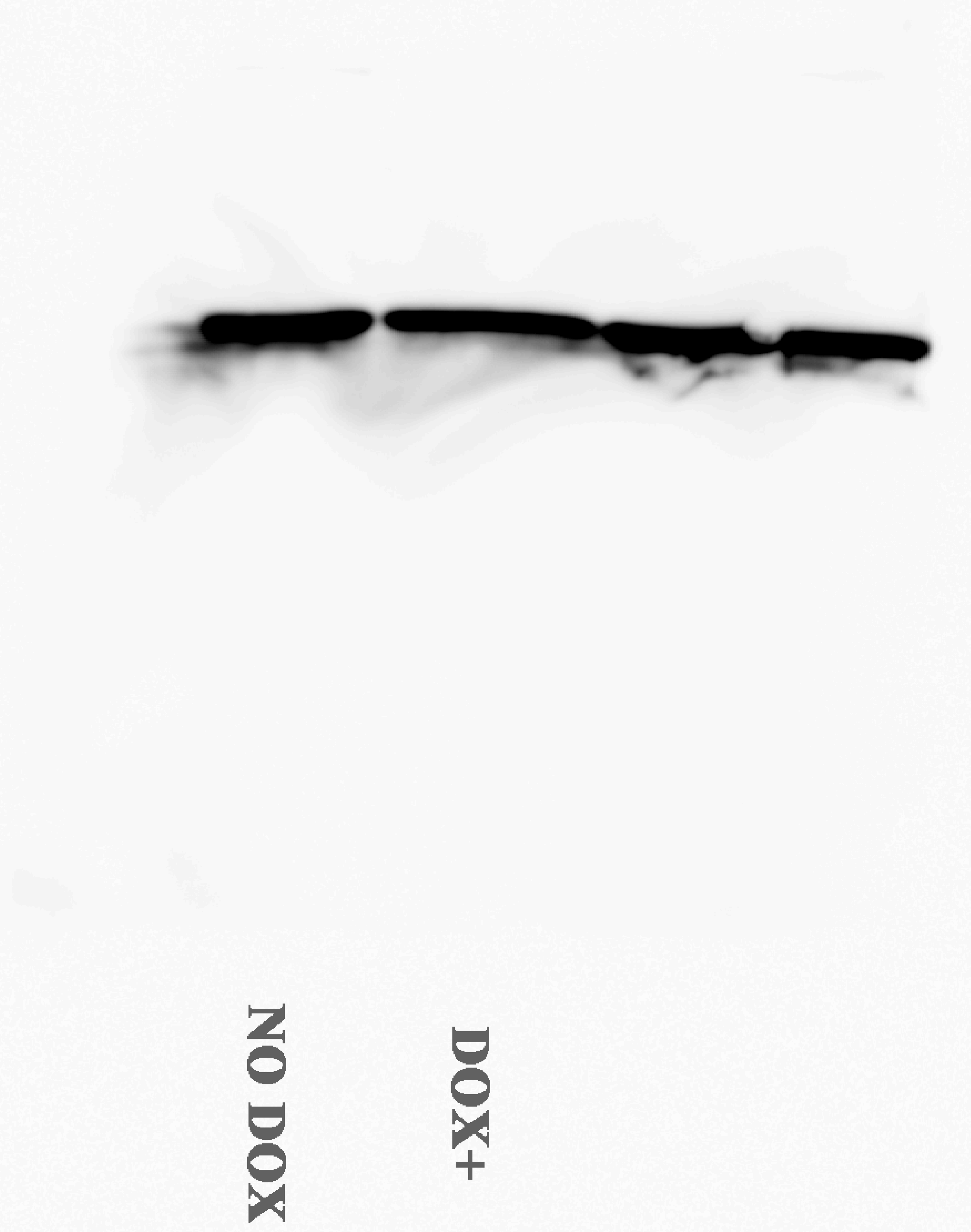

Fig 1G_GAPDH_4

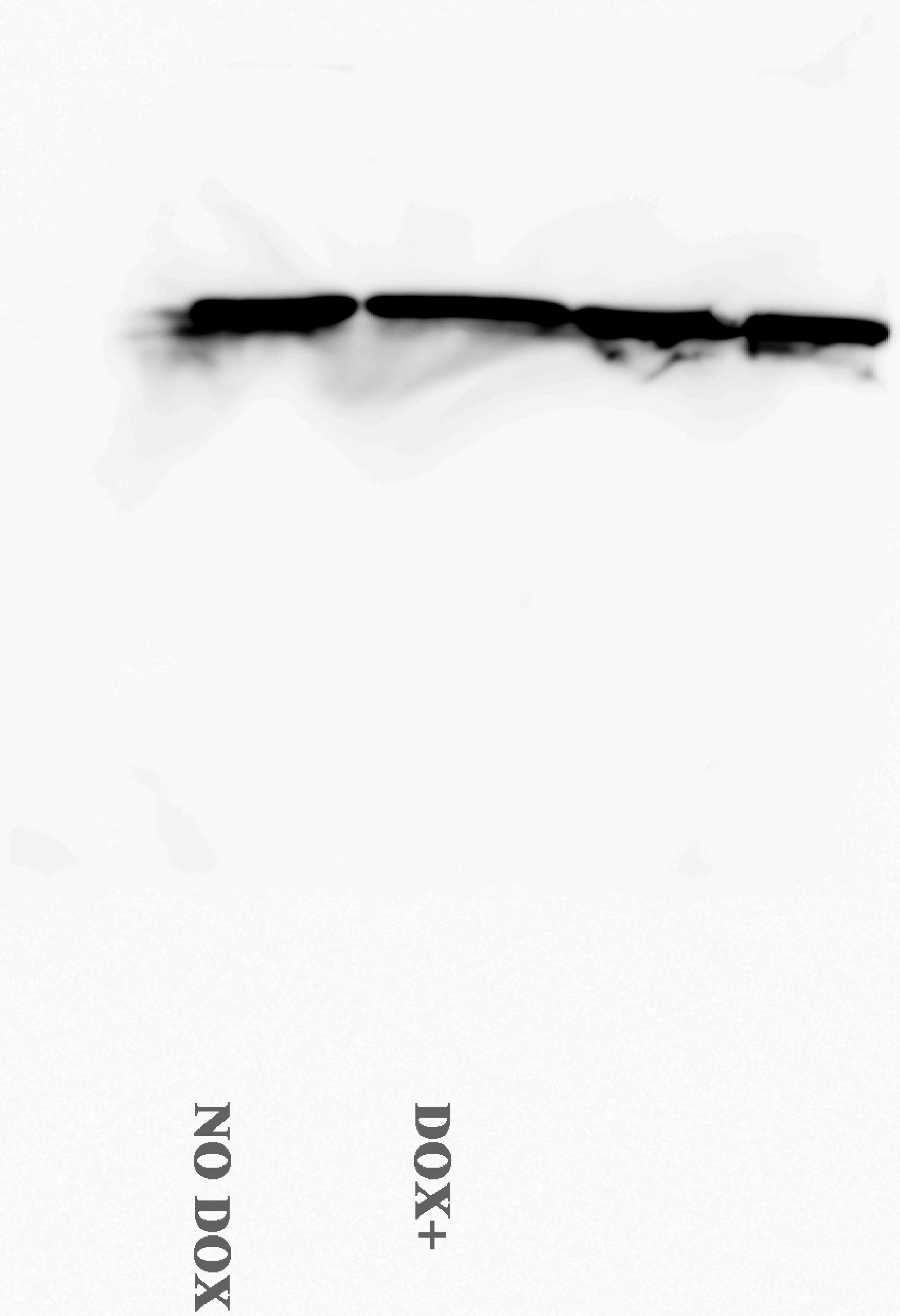

Fig 1G_GAPDH_5

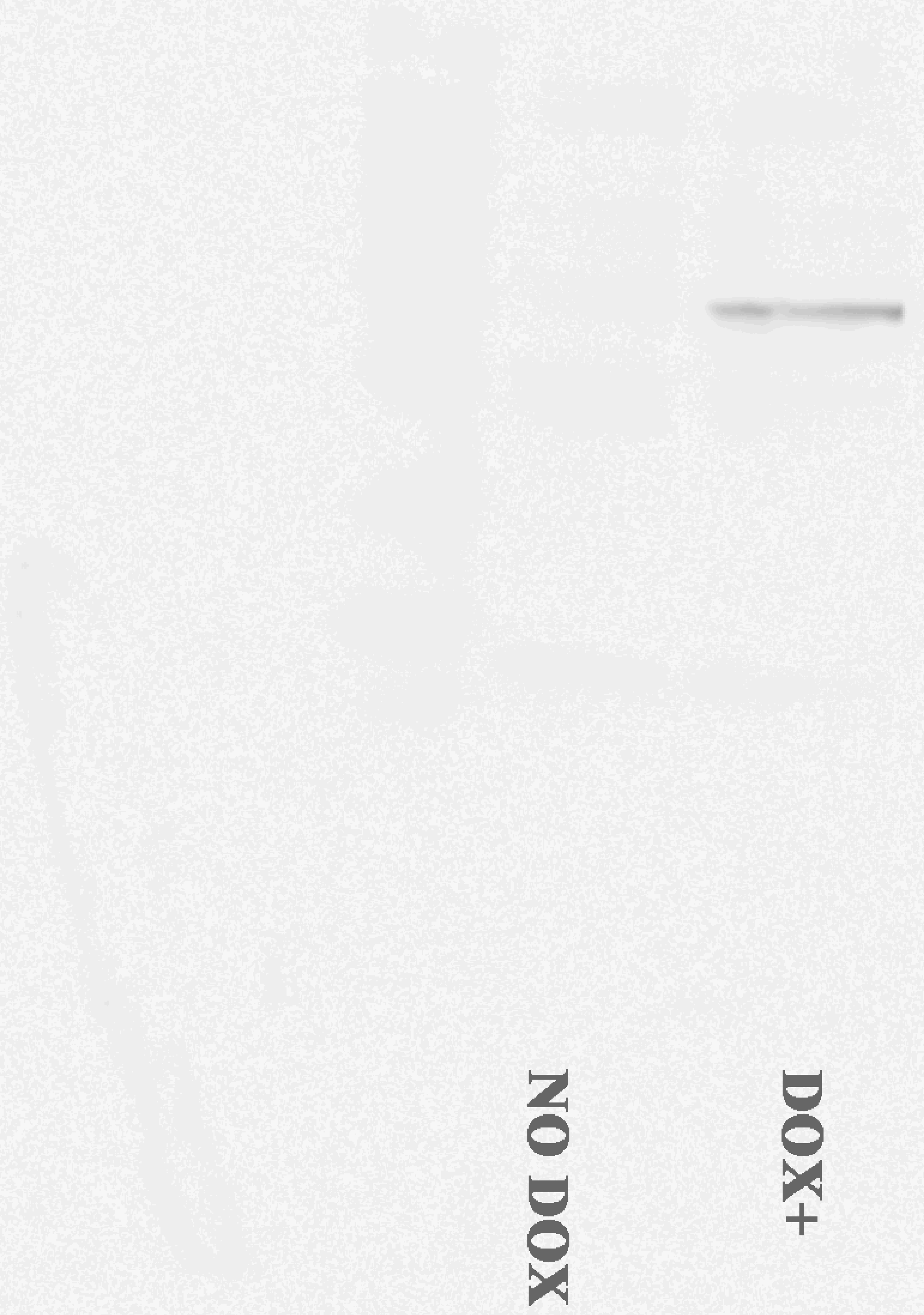

Fig 1G_P18_1

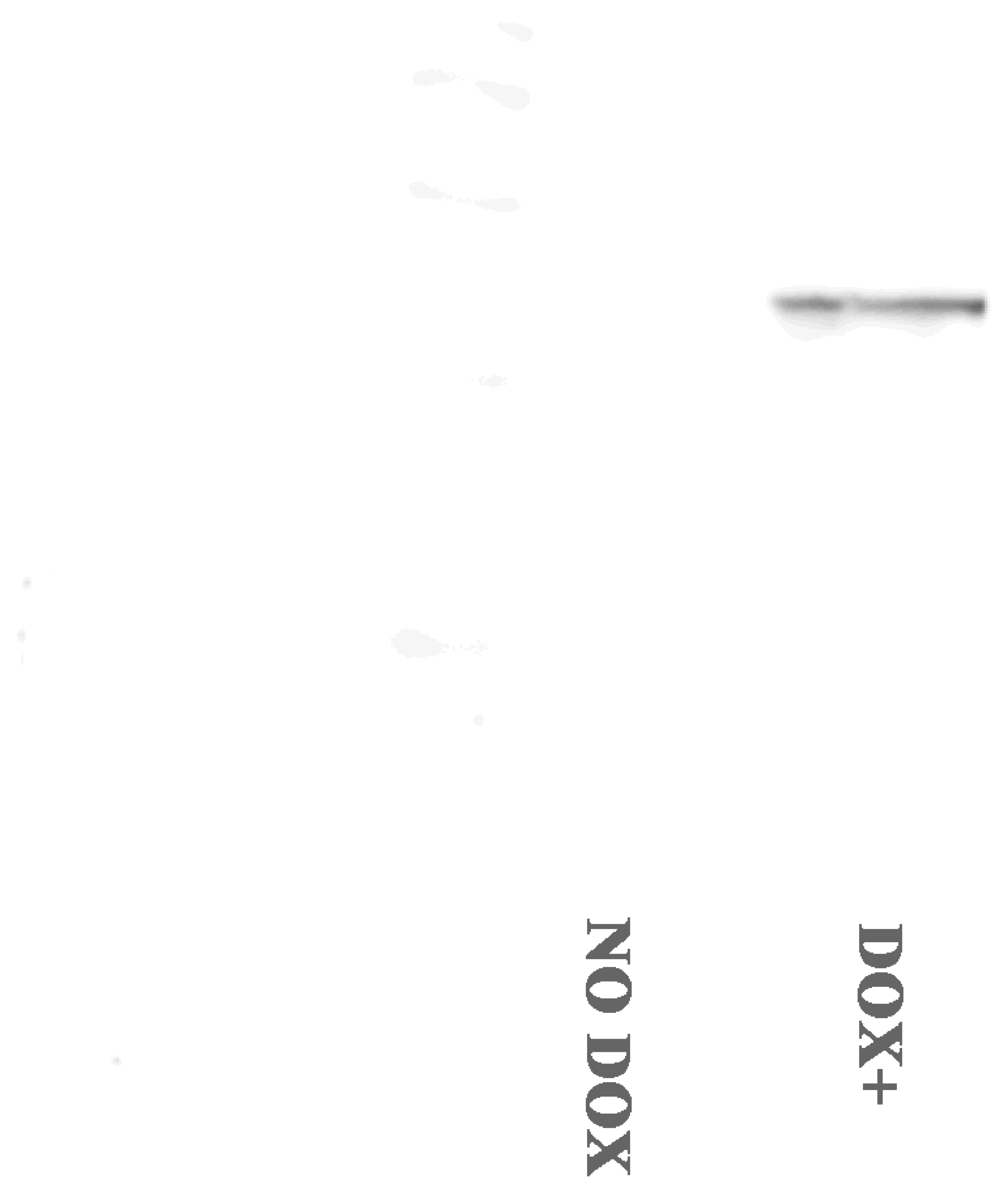

Fig 1G_P18_2

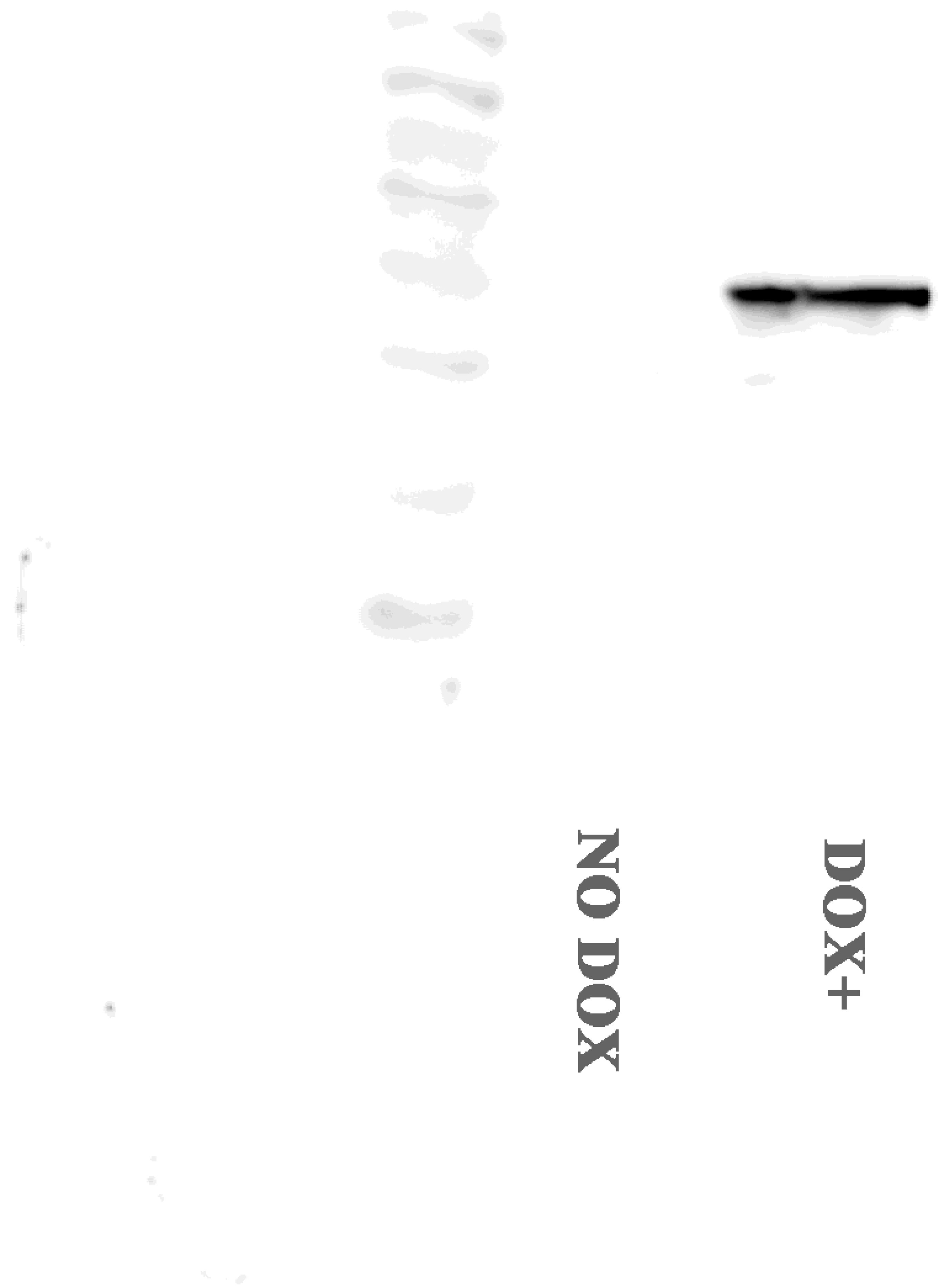

Fig 1G_P18_4

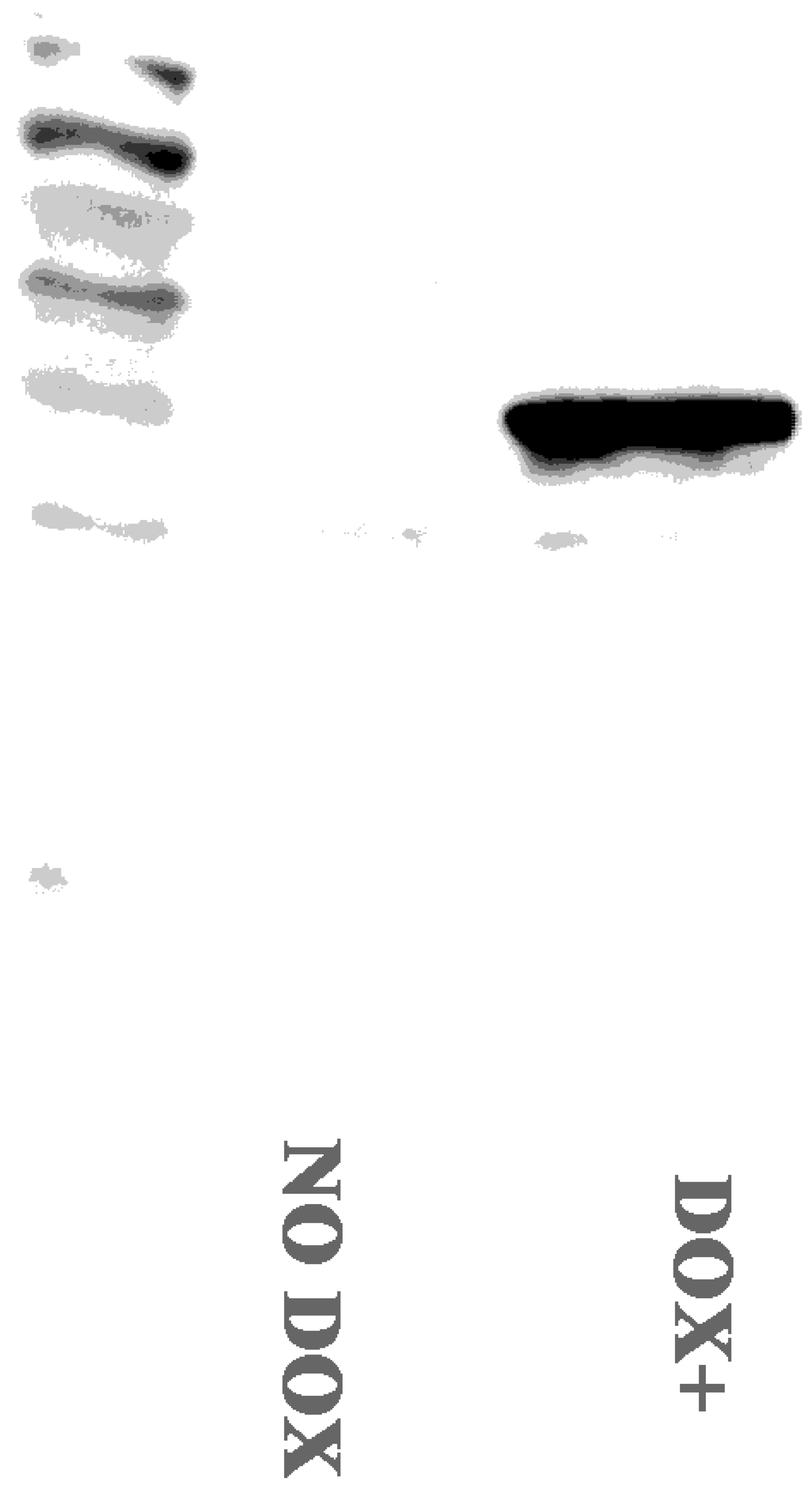

Fig 1G_P18_16

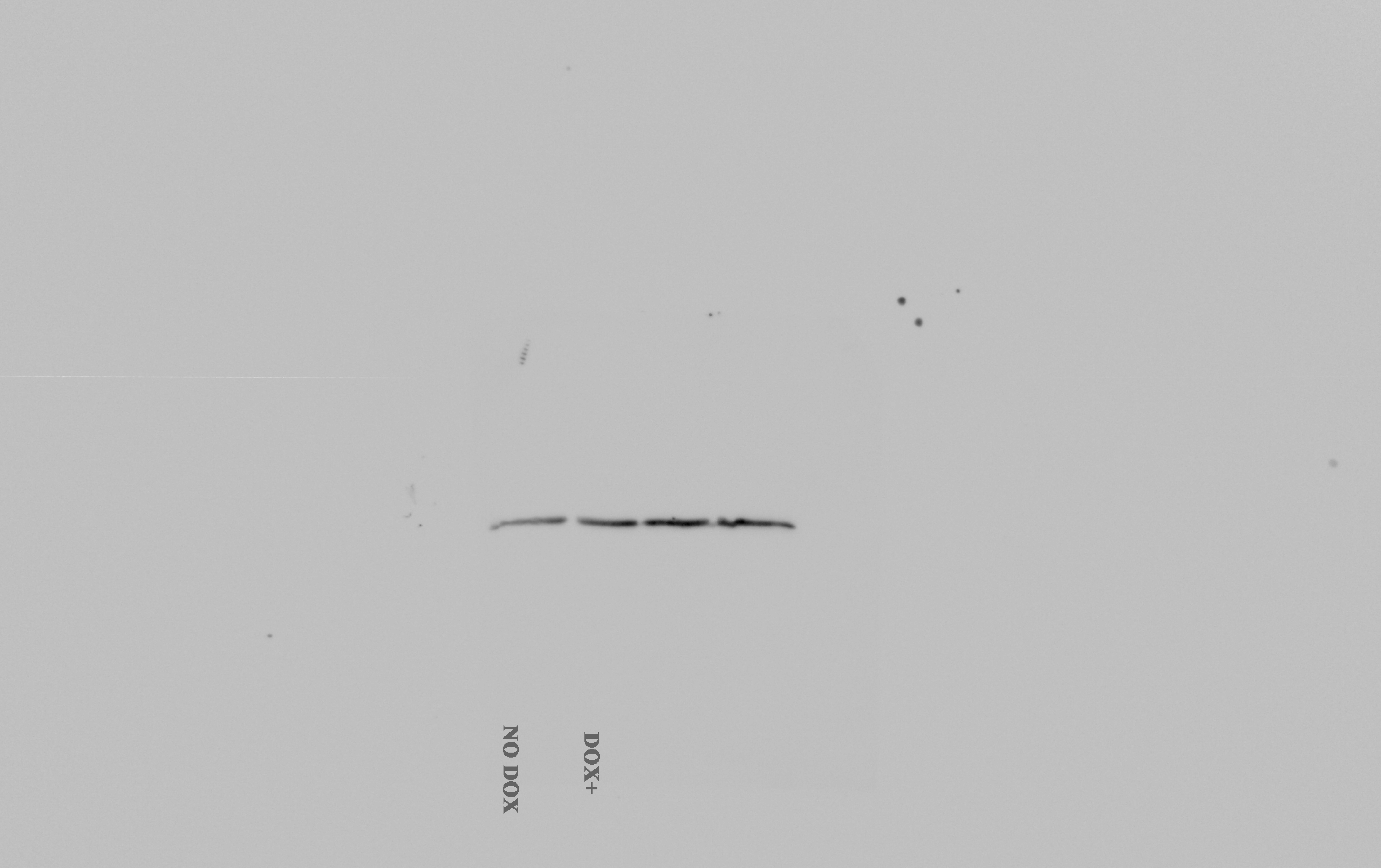

Fig 1H_gapdh_1

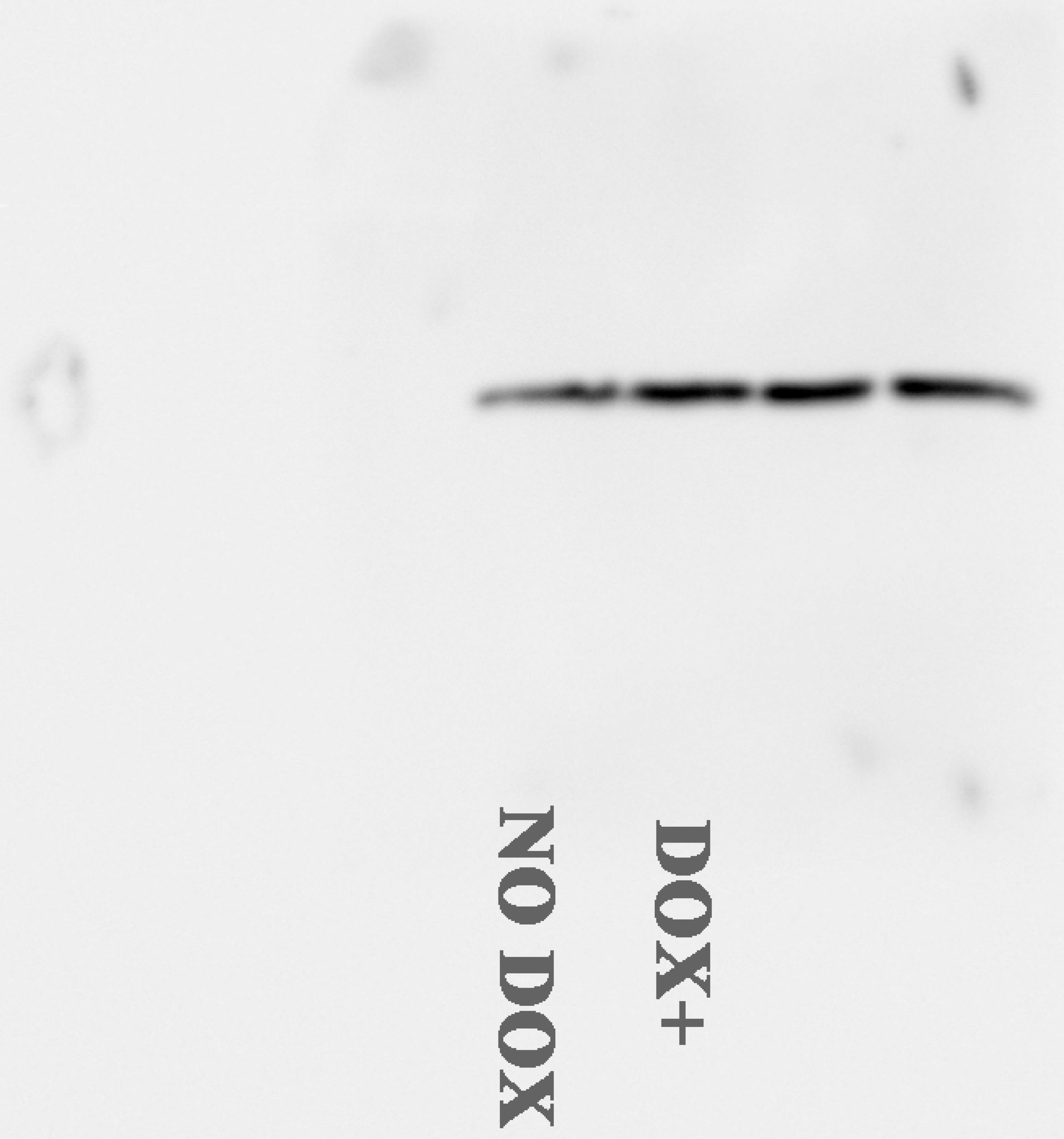

Fig 1H_gapdh_2

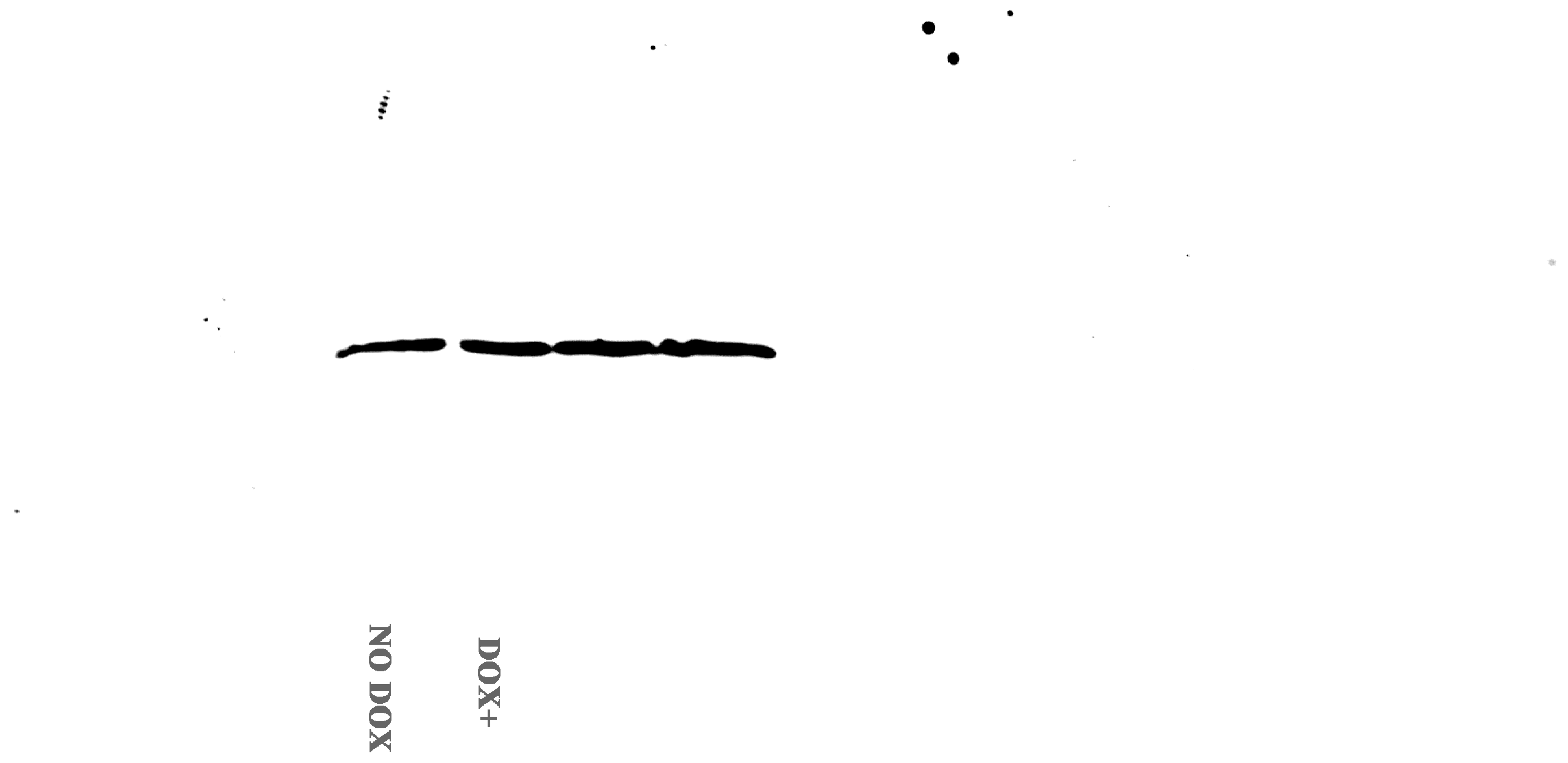

Fig 1H_gapdh_3

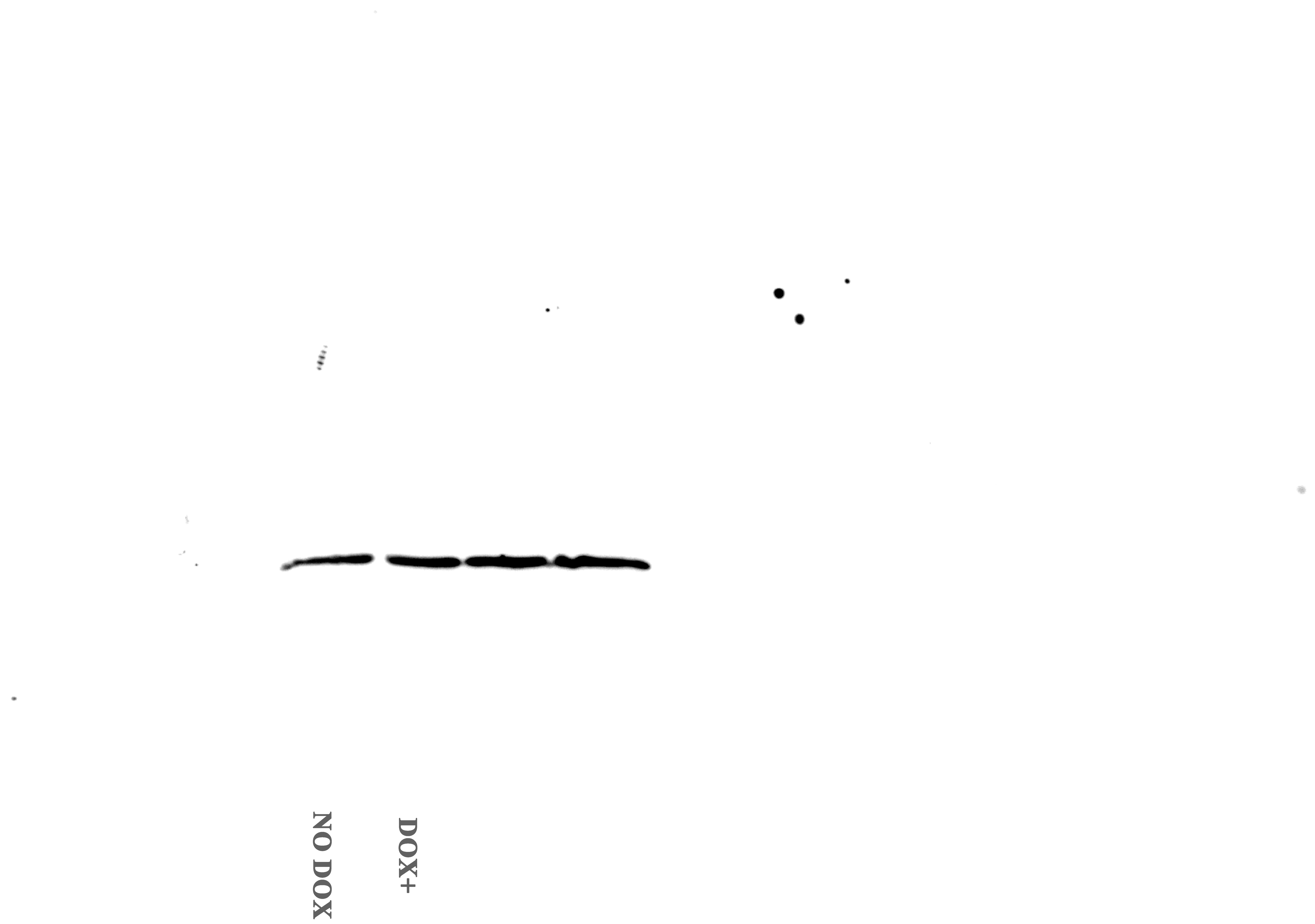

Fig 1H_gapdh_4

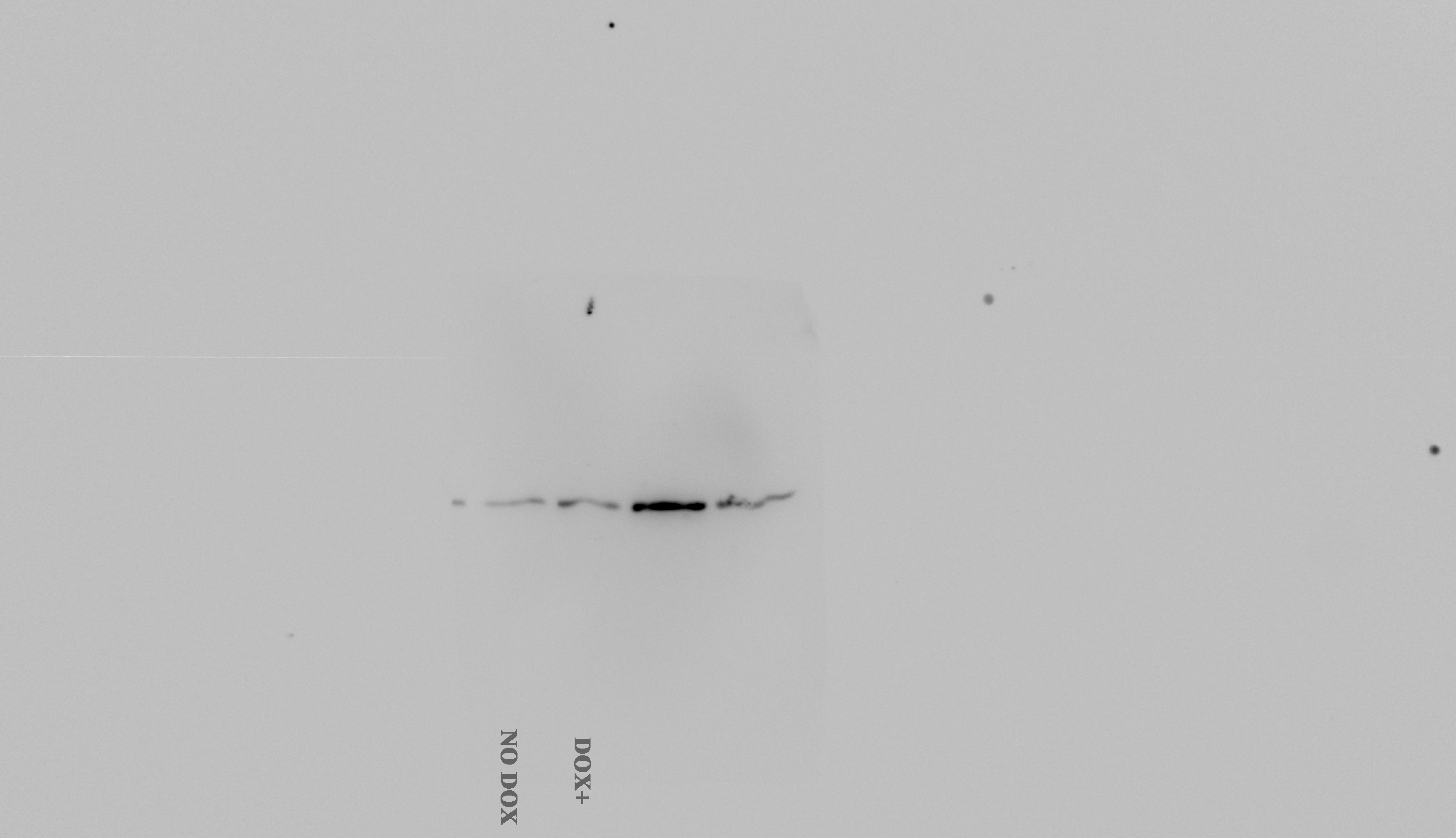

Fig 1H_nanog_1

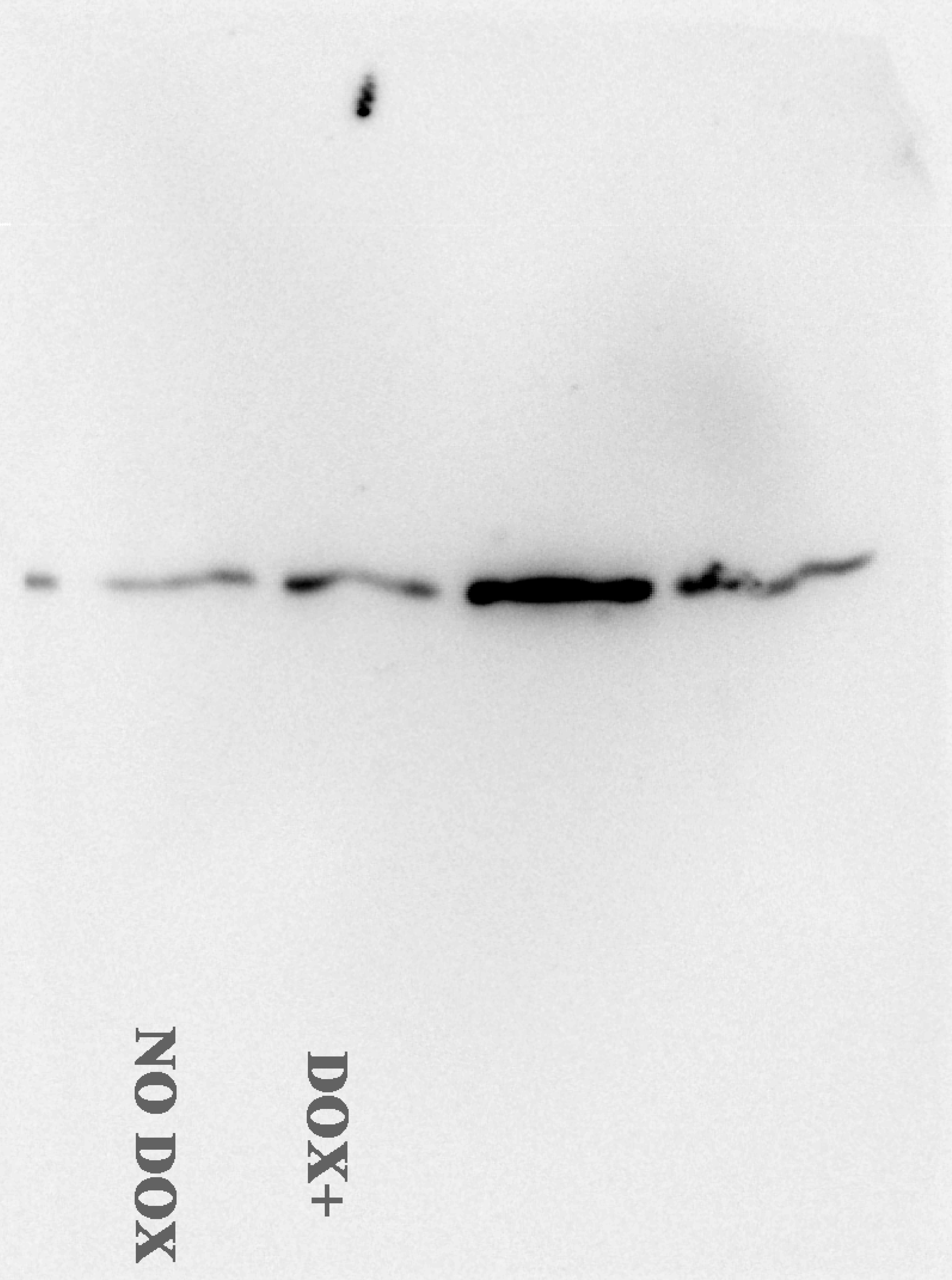

Fig 1H_nanog_2

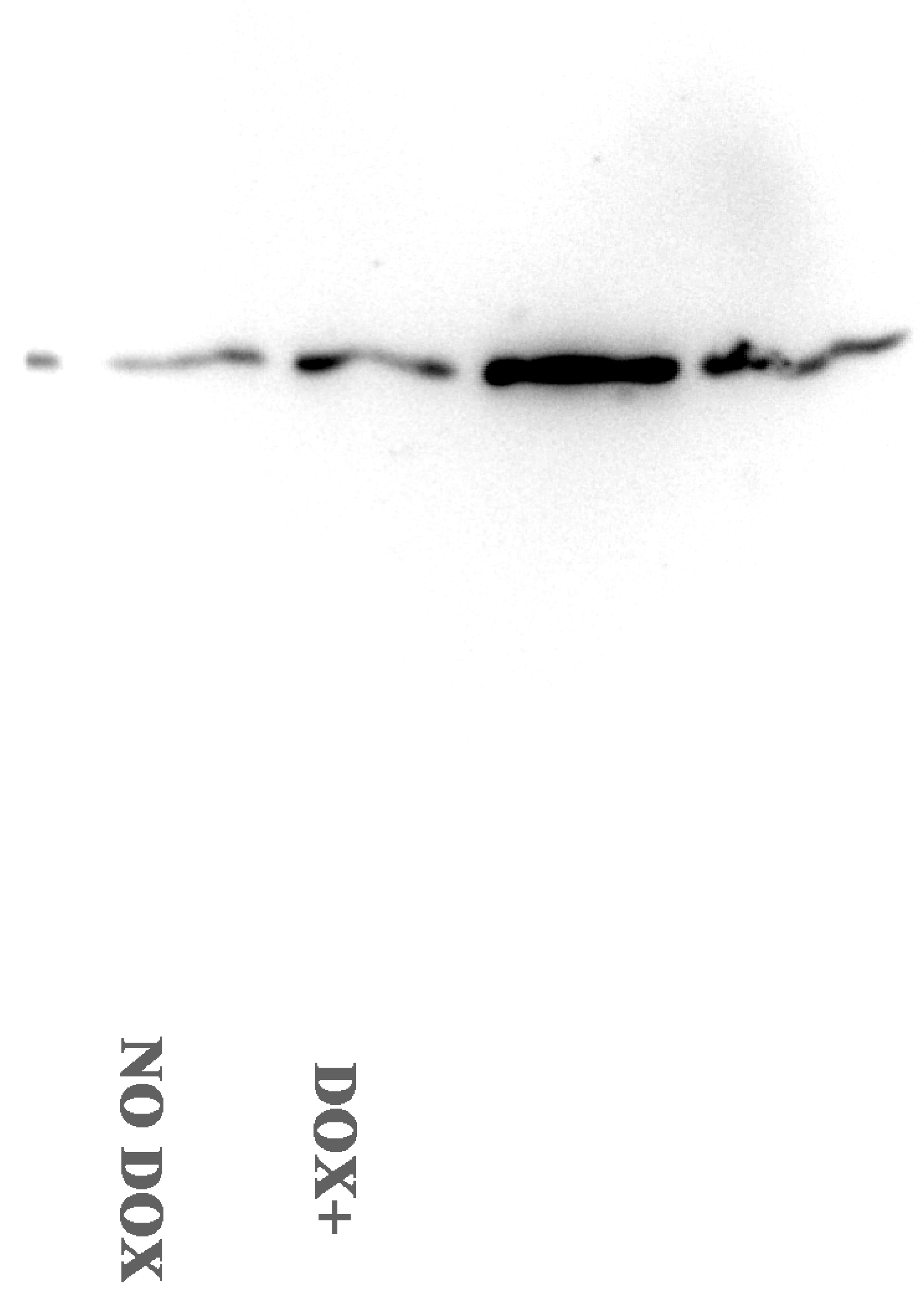

Fig 1H_nanog_3

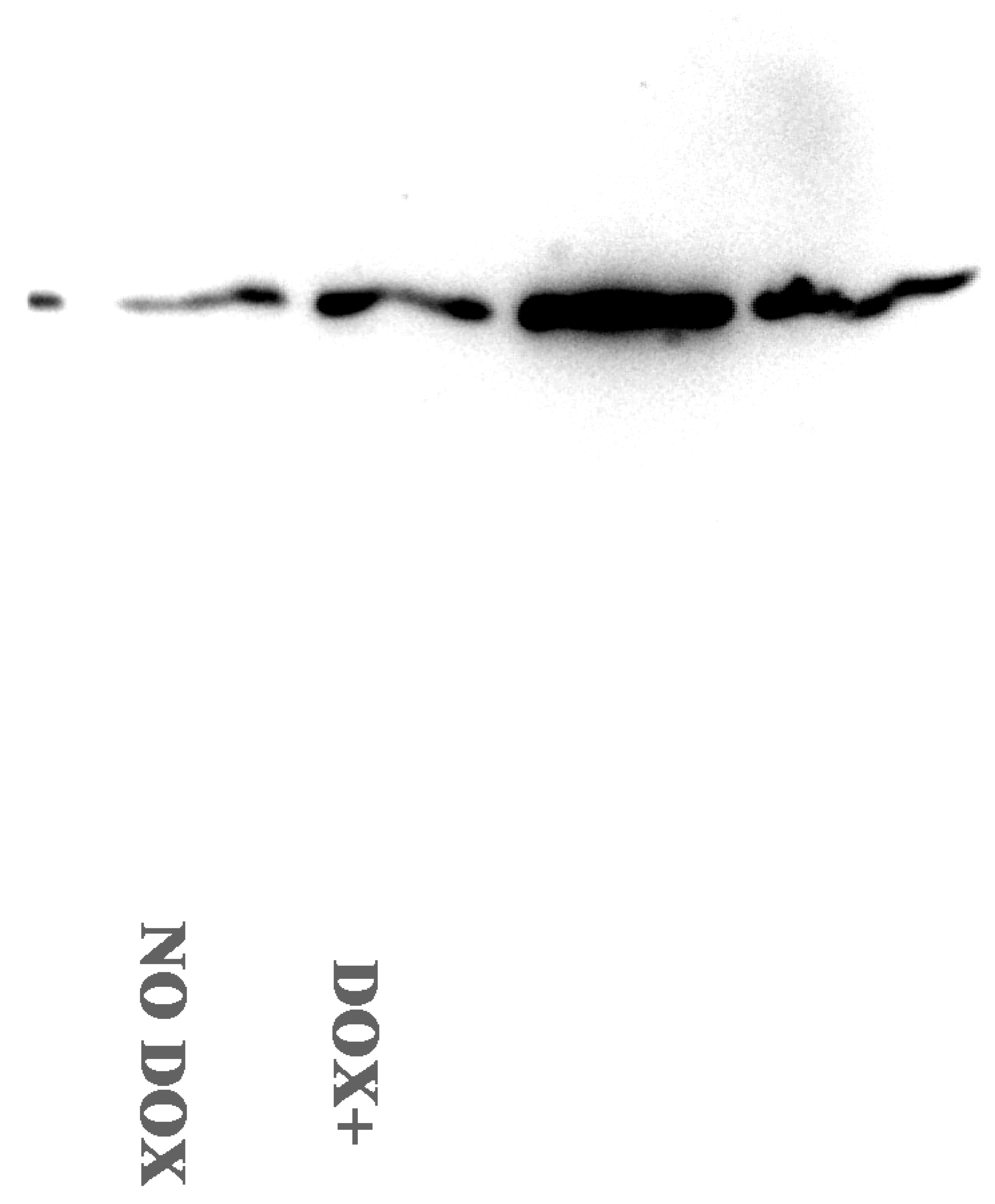

Fig 1H_nanog_4

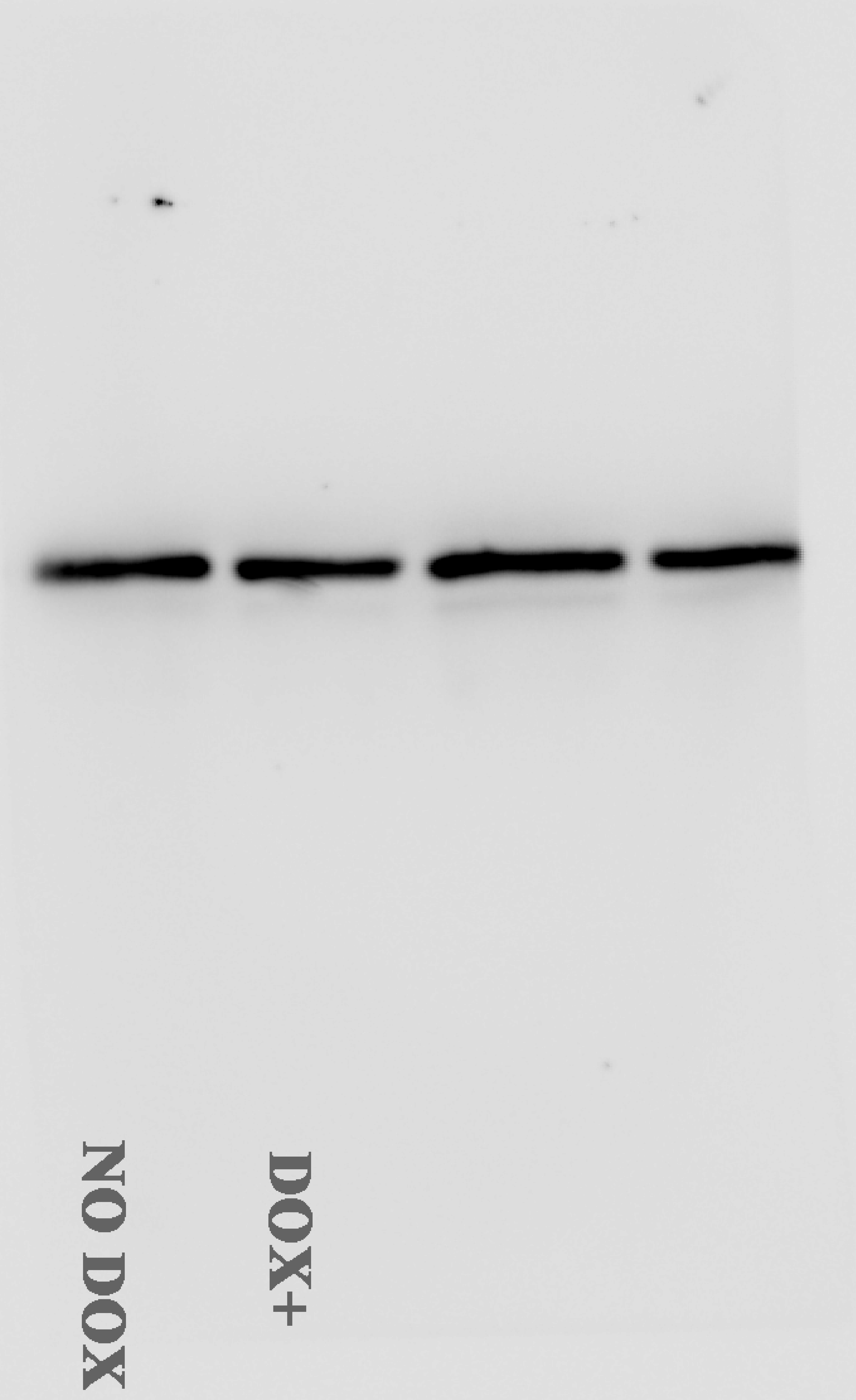

Fig 1H_oct4_1

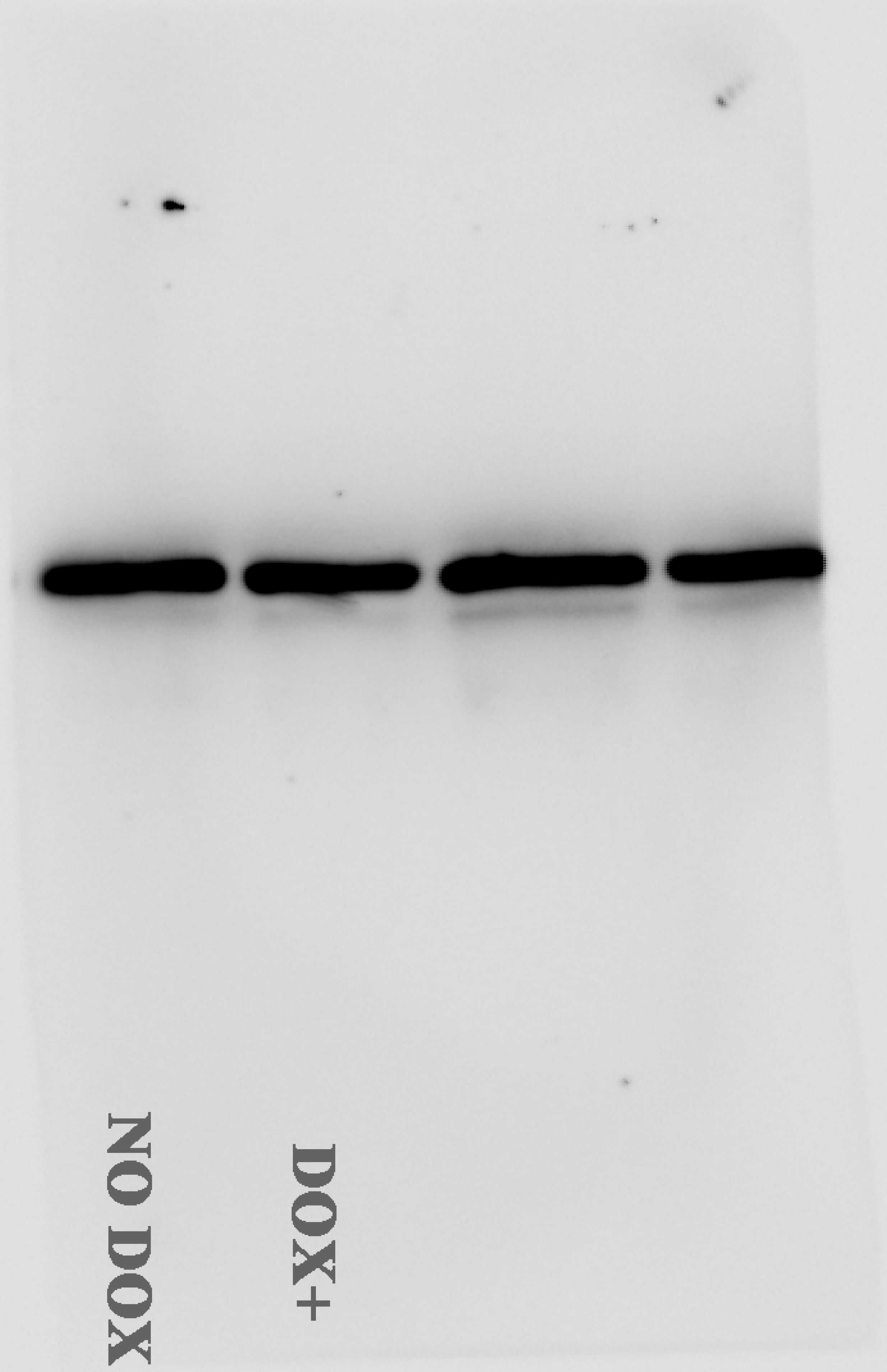

Fig 1H_oct4_2

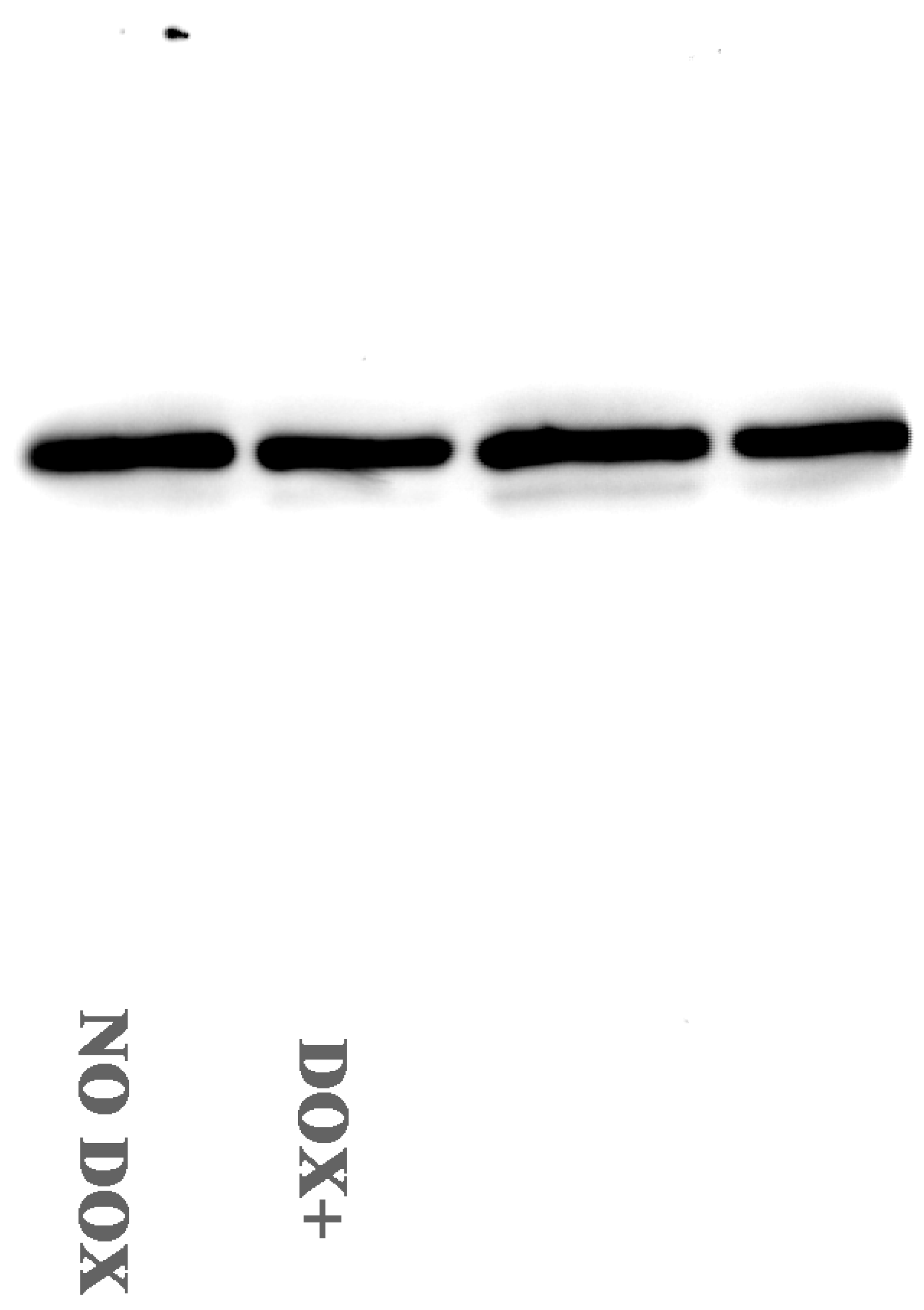

Fig 1H_oct4_3

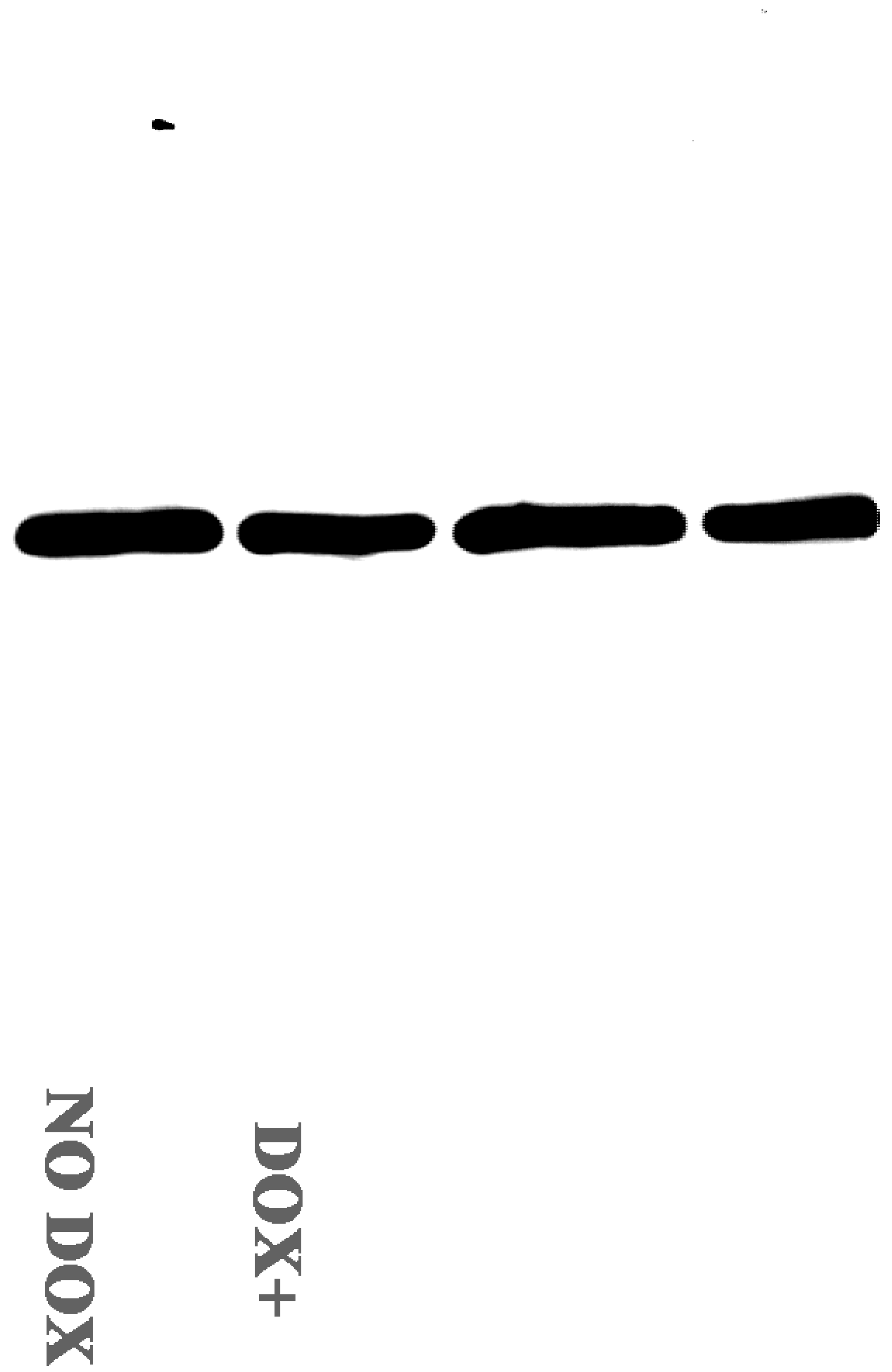

Fig 1H_oct4_4

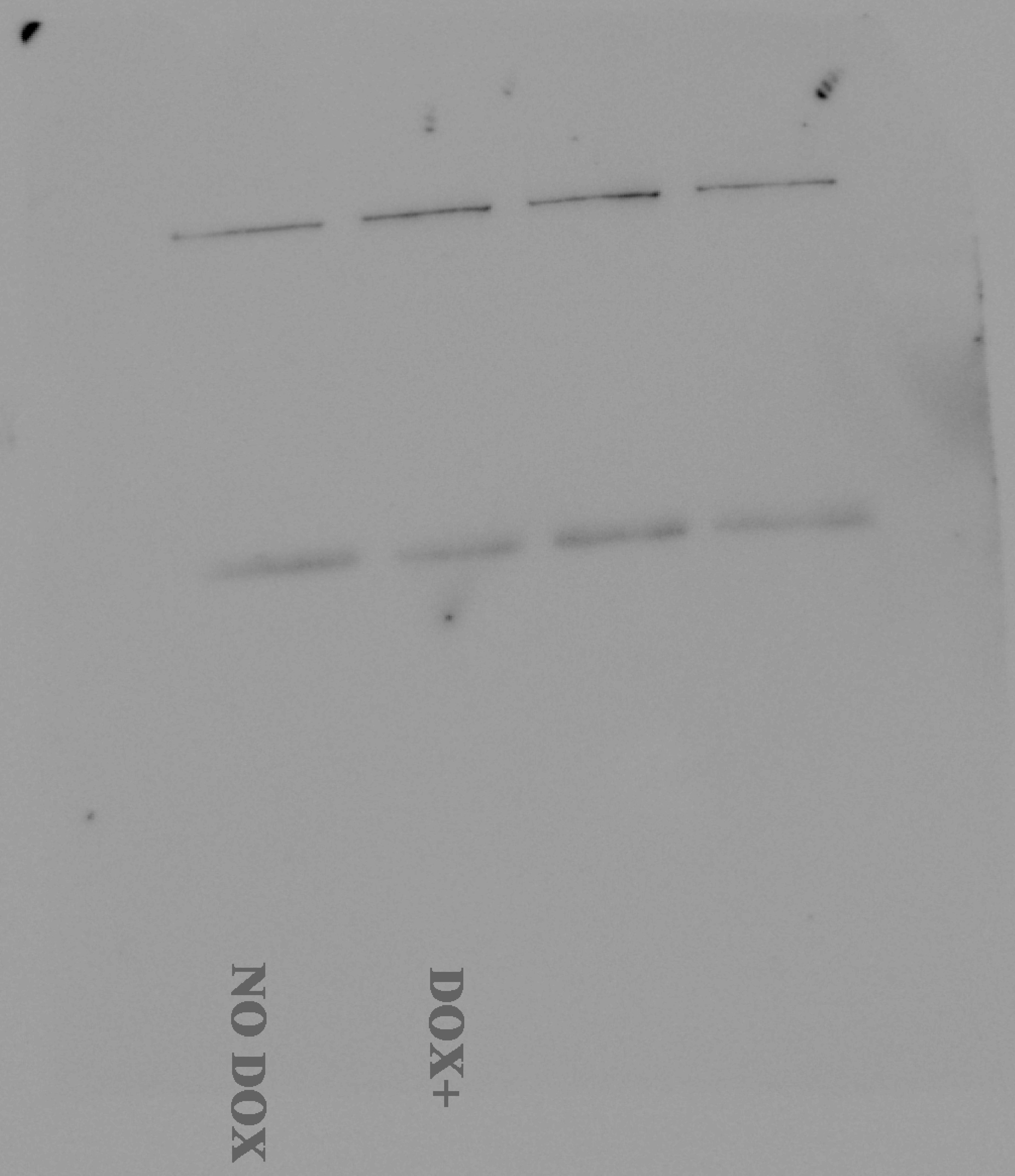

Fig 1H_sox2_1

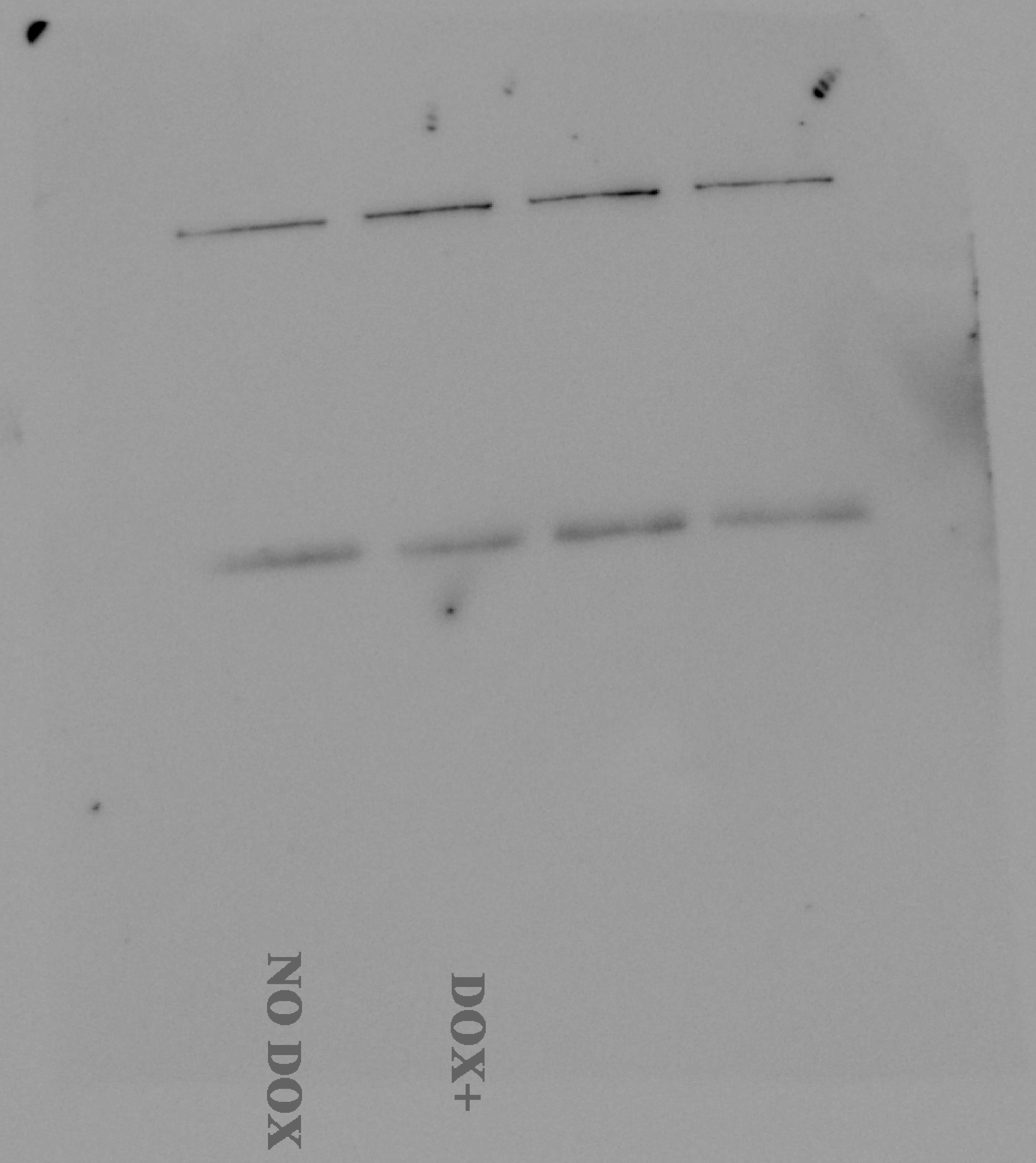

Fig 1H_sox2_2

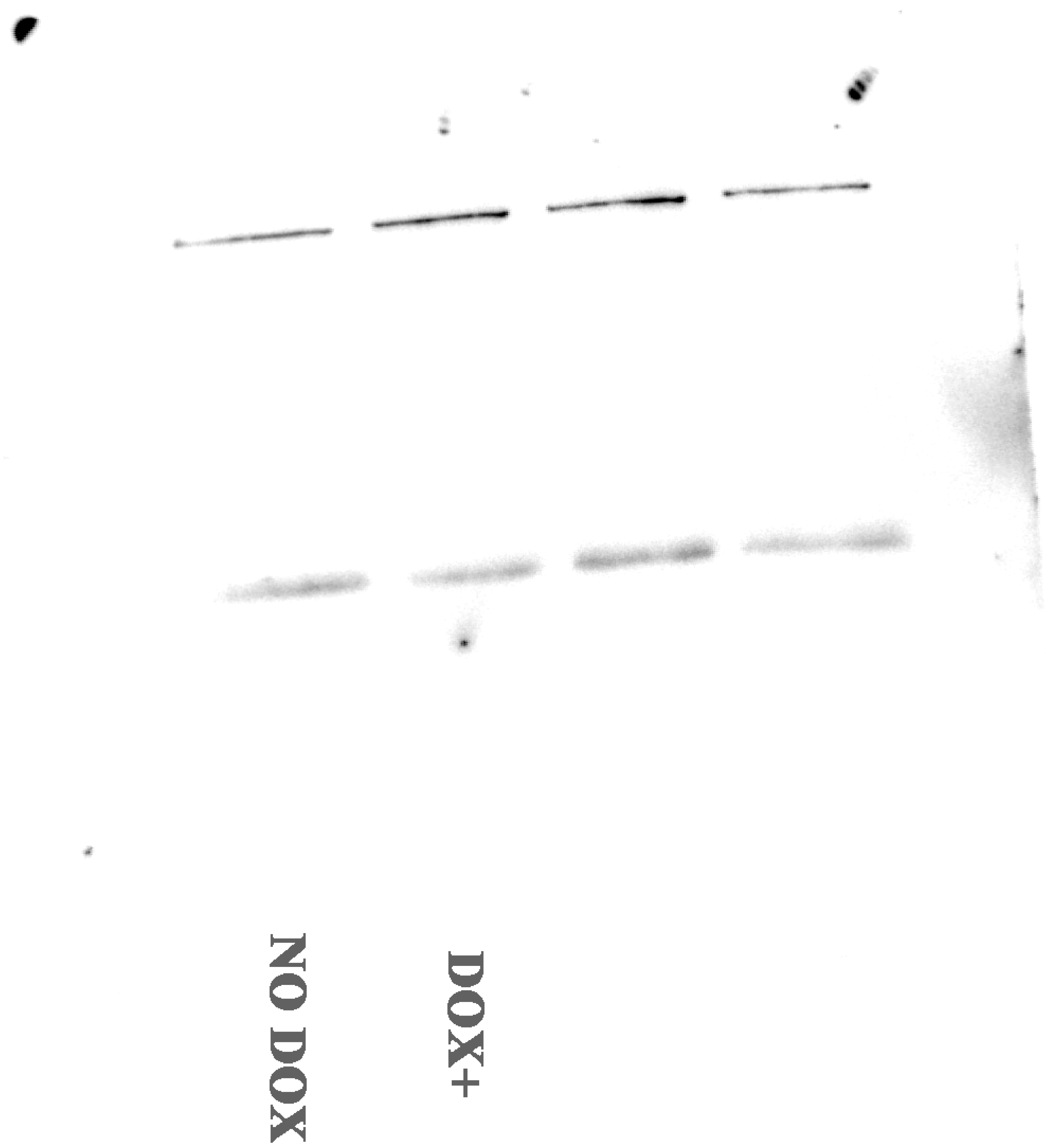

Fig 1H_sox2_3

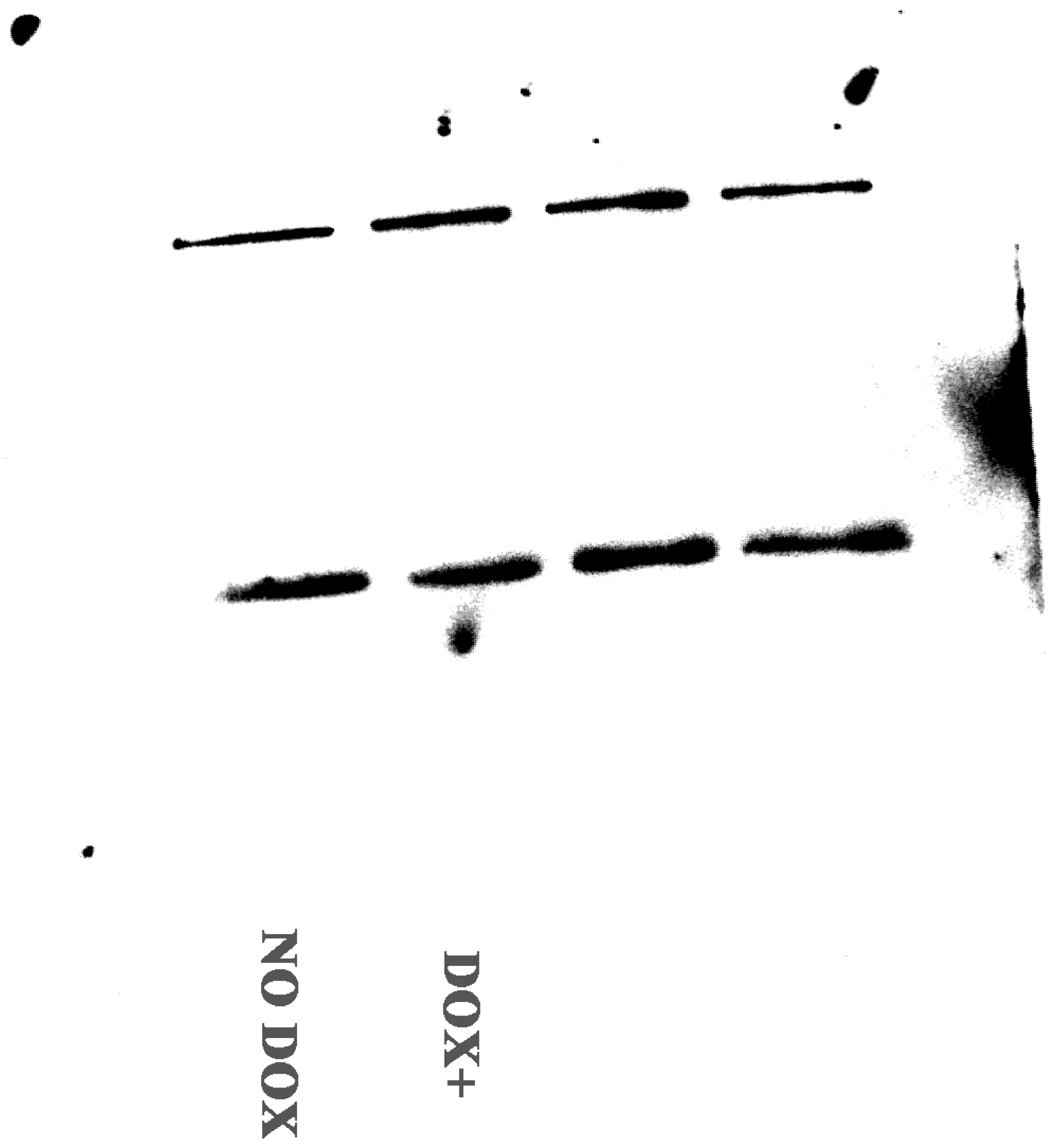

Fig 1H_sox2_4
